# Under-five mortality in the Rongo Sub-County of Migori County, Kenya: Experience of the Lwala Community Alliance 2007-2017 with evidence from a cross-sectional survey

**DOI:** 10.1371/journal.pone.0203690

**Published:** 2018-09-07

**Authors:** Joseph R. Starnes, Liz Chamberlain, Staci Sutermaster, Mercy Owuor, Vincent Okoth, William Edman, Troy D. Moon

**Affiliations:** 1 School of Medicine, Vanderbilt University, Nashville, Tennessee, United States of America; 2 Lwala Community Alliance, Lwala, Migori County, Kenya; 3 Vanderbilt Institute for Global Health, Vanderbilt University, Nashville, Tennessee, United States of America; TNO, NETHERLANDS

## Abstract

**Introduction:**

Childhood mortality remains a pressing problem in rural Kenya, and reducing under-five deaths is a key target of the Sustainable Development Goals. We aim to describe the reduction in under-five mortality in a rural Kenyan community served by the Lwala Community Alliance and factors associated with under-five mortality in this community.

**Methods:**

A cross-sectional survey containing a complete birth history was administered to a representative sample of the catchment area of the Lwala Community Alliance. Survival analysis techniques were used to describe temporal trends and risk factors related to under-five mortality.

**Results:**

1,362 children were included in the study, and 91 children died before the fifth birthday. The most common causes of death among children under five were malaria (19%), respiratory infection (13%), and anemia (11%). The under-five mortality rate was 104.8 per 1,000 live births from 1999 to 2006 and 53.0 per 1,000 after the founding of the Lwala Community Alliance in 2007. Factors associated with under-five mortality included year of birth (HR 0.931; 95% CI: 0.877, 0.988; p = 0.019), multiple-gestation pregnancy (HR 6.201; 95% CI: 2.073, 18.555; p < 0.001), and birth in the long rain season (HR 1.981; 95% CI: 1.350, 2.907; p < 0.001). Birth spacing greater than 18 months was negatively associated with under-five mortality (HR 0.345; 95% CI: 0.203, 0.587; p < 0.001).

**Conclusions:**

There was a significant decrease in under-five mortality before and after the presence of the Lwala Community Alliance. Multiple-gestation pregnancies, birth season, and short birth spacing were associated with under-five mortality and provide possible targets to further reduce mortality in the region. This provides both hyper-local data necessary for implementation efforts and generalizable data and sampling methods that may be useful for other implementing organizations in sub-Saharan Africa.

## Introduction

The international community has acknowledged children’s right to life for more than 25 years [1]. The Millennium Development Goals (MDGs) set an aggressive target of reducing under-five mortality (U5M) by two-thirds between 1990 and 2015 [2]. The world attained large gains over this time period, including an overall decrease of 52% in U5M with similar gains among older children [3,4]. With the transition to the Sustainable Development Goals (SDGs), the United Nations moved towards an absolute goal of ending preventable child deaths and reducing overall U5M to less than 25 per 1,000 live births by 2030 [5].

Kenya saw slightly smaller gains in U5M over this time period but still made large strides [3]. However, regional variability within Kenya is striking. The overall U5M rate was 50.8 per 1,000 live births in 2015, but county-level estimates ranged from 20 per 1,000 live births to well over 100 [3,6]. This wide variation in within-country metrics is consistent with data from varying regions [7–10]. It has been suggested that the focus of the MDGs on national data ignored these within-country inequities [9,11–13], and that increased health spending often benefits those that were best off to start [11,12,14]. These subnational differences within countries highlight the need for local data to inform health services implementation efforts [15–17].

Well-studied and cost-effective interventions are available to reduce child mortality [18]. Scaling of existing interventions could reduce neonatal deaths by 71% at a cost of less than $2,000 per life saved [19,20]. Interventions include measles vaccination and vitamin A supplementation [21], rotavirus vaccination [22], zinc treatment for diarrhea [23], antibiotic treatment for dysentery [24], oral rehydration salts for diarrhea [25], insecticide-treated bednets [26], and a host of other low-cost interventions [19]. Implementation of such programs requires country-led, data-driven processes that engage the community [27]. Community Health Workers (CHWs) can help engage the community and overcome healthcare provider shortages to reduce child mortality and have been used successfully in rural western Kenya [28–30]. The most effective ways in which to engage these CHWs to improve healthcare outcomes is an important research priority in childhood mortality [31].

Data from rural western Kenya suggest that malaria, acute respiratory infection, anemia, and diarrhea are the most common causes of death in children under five [32,33]. These are consistent with data from international studies of childhood mortality and regional studies on the most common causes of child hospitalization [3,33,34]. Rates of under-five death generally decreased each year between 2003 and 2010, although there was an increase in 2008 possibly associated with election-related unrest and resultant supply chain difficulties [32,35]. Factors associated with child death outside of cause of death have been less well investigated locally, although it is known that the majority of deaths occur at home and that antenatal care is of variable quality [33,36]. Previous studies elsewhere have found a host of factors associated with child mortality in sub-Saharan Africa (SSA), including short birth intervals [37–39], poor access to healthcare [38,40], poor health seeking behavior [39], home birth [41], maternal age [38], village and ethnic group [38], low maternal education [42], high birth order [39], multiple–gestation pregnancies [38,43], season of birth [38], livestock ownership [44], indoor smoke [45], and living in a rural setting [46].

**Fig 1:**
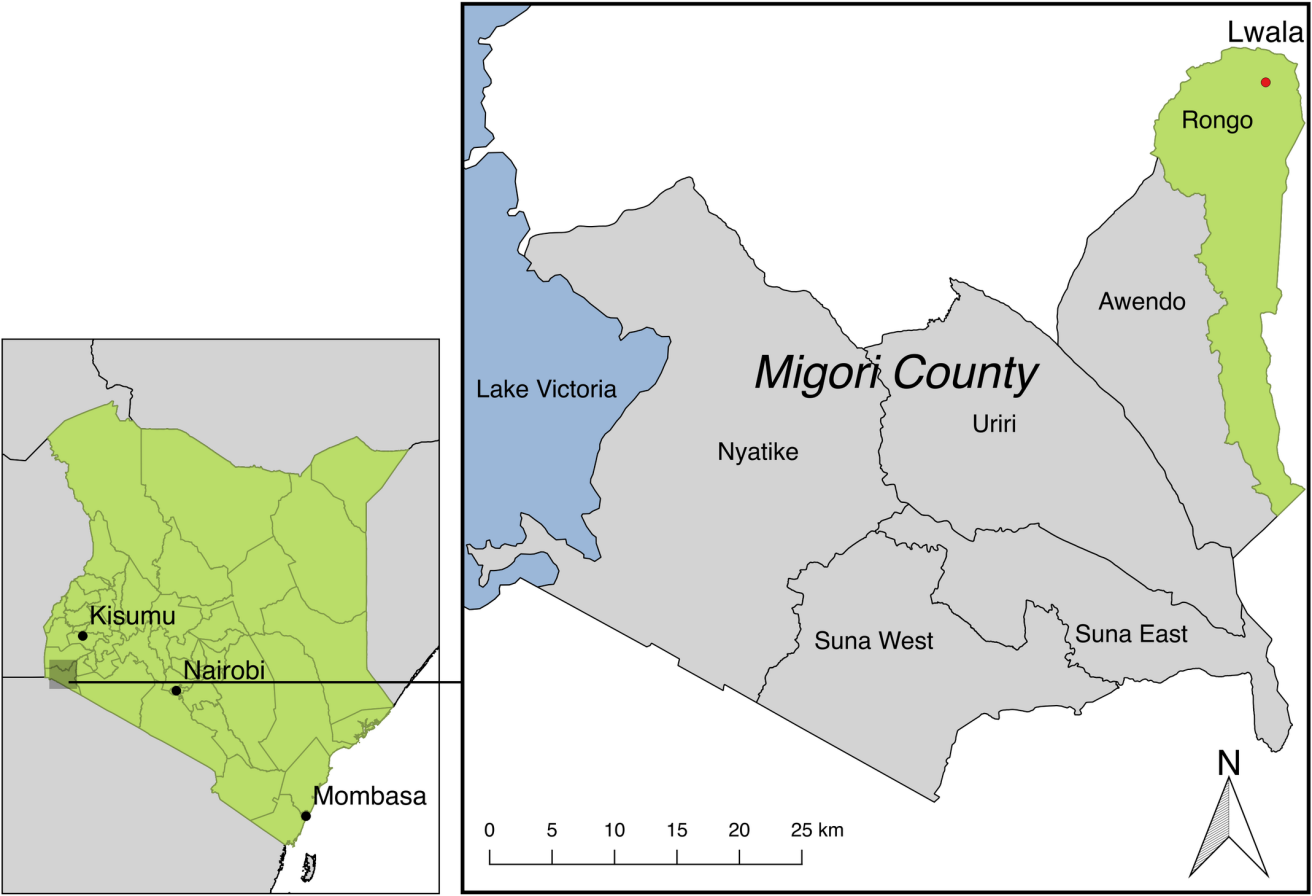
Migori County, Kenya. Kenya with Migori County framed (left). Migori County with six divisions labeled (right). Lwala is located in the northeast corner of Rongo sub-county near Lake Victoria. The closest major city is Kisumu. This map was created using data from the Database of Global Administrative Areas.

The Lwala Community Alliance (LCA) is a community-led, multifaceted organization in rural western Kenya working with a community of 30,000 people to advance their own comprehensive wellbeing. Founded in 2007, LCA operates a hospital and clinic with inpatient, outpatient, maternal, and HIV care, as well as an innovative CHW program incorporating traditional birth attendants. An integrated maternal and child health program, entitled Thrive Through 5, attempts to enroll every pregnant woman and child born in the catchment area. LCA also has programming in education, economic development, and public health. Similar integrated models have shown promise in rapidly decreasing child mortality [47]. LCA operates in the Rongo sub-county, one of six official divisions of Migori County (Fig 1). Migori County, formerly part of Nyanza province, is near Lake Victoria and has an economy primarily reliant on subsistence farming [48]. The U5M rate in Nyanza was 82 per 1,000 live births in 2014, which was the highest among all regions in Kenya [6].

In 2017, LCA implemented a community-wide survey, designed to be representative of the entire surrounding catchment area, to better understand priority health metrics of the communities LCA serves. This survey provides both hyper-local data necessary for LCA´s implementation efforts as well as generalizable data and sampling methods that may be useful for other implementing organizations working in sub-Saharan Africa.

## Methods

### Study setting

The study was conducted in the North Kamagambo location in the Rongo sub-county, which corresponds to the geographic catchment area of LCA. The population of the catchment area is estimated to be 30,000 among a population of approximately 920,000 in the greater Migori County [49]. The catchment area relies primarily on subsistence farming. Formal healthcare services in the catchment area are provided by LCA and two government-run facilities. LCA is a level four district hospital while the government-run facilities are level two dispensaries [50].

### Sampling

Sample size was determined using U5M as a binary outcome using a binomial test to compare one proportion to a reference value. Using a power of 0.85 and alpha of 0.05, a sample size of 500 was needed to detect a difference of 40 per 1,000 live births from the rate of 82 per 1,000 live births in Nyanza [6]. This assumed just one birth per household surveyed, which was very conservative.

A proportional stratified sampling method was used to give all households equal chance of selection despite differing population densities. The coordinates of all 5,930 households in the catchment area were input into mapping software. Because we used this method for sampling, a household was defined as a house structure. The catchment area was then broken into 11 grid squares based on the number of survey days. The number of households needed in each grid square to reach a total of 500 was determined to be 8.4% of households within each square. On each survey day, the team travelled to the geographic center of a square. A random degree between 0 and 360 was then generated for each interviewer as well as a random series of skip numbers. The interviewer then travelled along the line determined by the degree and visited the appropriate houses using the skip numbers to determine which houses were interviewed. The survey was administered to a random resident over the age of 18 in each household. The two lowest density squares were surveyed on the same day, yielding a total of 10 days of data collection. This sampling method has been described previously, and we have minimized the bias associated with beginning in the center of a town by using the center of an arbitrary geographic grid square [51].

### Survey

The survey contained more than 270 questions across multiple domains and was modeled on validated tools, including the Demographic and Health Survey [6] and the Strengthening Communities through Integrated Programming survey previously used in Mozambique [52–55]. The survey was designed to obtain population-based data for indicators across multiple areas, including child mortality, vaccination, family planning, HIV, water and sanitation, mental health, economics, and education (S1 Appendix). Included in the survey was a complete birth and death history of all children born in the last 18 years in the households of those surveyed. Gender, relationship to the respondent, age, and cause of death (if applicable) were captured for each of these children. Cause of death was directly reported by respondents in a multiple-choice format with an other option for causes not covered by existing answers. Demographic, health, and socioeconomic data were captured about the respondent and household. The survey was piloted with small groups of respondents to ensure applicability and comprehension.

Surveys were administered by trained interviewers not otherwise employed by LCA. All were hired from the community and were fluent in English, Dholuo, and Swahili. Surveys were administered on tablets using a customized CommCare (Dimagi, Cambridge, MA) form to reduce data entry errors and ensure appropriate skip logic. Data collection occurred in January 2017 over a two-week period.

### Study population

All children born after January 1, 1999 for whom the respondent was the mother or father were included in the analyses. There were 1,362 children who met these criteria. Among these children, 91 died before their fifth birthday.

### Ethics approval and consent to participate

This study was approved by the Ethics and Scientific Review Committee at Amref Health Africa (No. P290/2016) and the Institutional Review Board at Vanderbilt University Medical Center (No. 161396). Written informed consent was obtained from all participants. Approval was also obtained from the local area chief and Ministry of Health.

### Statistical analysis

A preliminary list of variables available in the survey was created based on previous studies of associations with childhood mortality [15,17,34,37–40,42–46,56,57]. Cox regression models with clustering at the household level were used to estimate the effects of independent variables on survival with effects given as hazards ratios. Observation time for each child began on the birth date and ended on whichever came first among the death date, the birthday marking the end of the risk period (first or fifth birthday depending on the model), and the interview date. An initial model with all 12 variables was trimmed to 9 based on collinearity and measurement of similar concepts to avoid overfitting. Child gender was not included in the final model to allow the inclusion of other variables, but an additional model with gender did not have substantially different hazard ratios or significance (S2 Appendix). The proportional hazards assumption was evaluated for all presented models using Schoenfeld residuals [58]. An initial U5M model containing the multiple-gestation pregnancy indicator violated the proportional hazards assumption because the effect of this variable is primarily in the first year of life. This is consistent with previous literature [38]. A time-varying indicator that turned to zero for all children at the time of the first birthday was used in the final model. Concordance for the final model was calculated using Harrell’s C [59,60].

A time-varying indicator was created to analyze time periods relative to the founding of LCA in 2007. This indicator was set to one on January 1, 2007. Kaplan-Meier curves were used to visualize mortality curves before and after this date, and the log-rank test was used to compare these curves. Mortality rates were calculated using the Kaplan-Meier cumulative failure function. All analyses were performed using Stata version 14.2 (StataCorp LP, College Station, TX).

## Results

### Descriptive statistics

The survey was administered to 539 respondents with a response rate of greater than 99%. 428 (79.6%) of these respondents were the mother or father of at least one child born in the last 18 years and were included in this analysis. Among these respondents, there were a total of 1,362 live births with 91 deaths before the fifth birthday. There was a slight female majority with 52% of births being female. There were between 50 and 100 births in each year between 1999 and 2016, and 7 children were born in the first month of 2017 prior to survey administration. There were no missing data with the exception of maternal age for 3 children (0.2%).

Table 1 shows descriptive statistics by vital status at the fifth birthday or the time of the interview. The median age of living children was 9 years, and the median age at death was 8 months. Deceased children were generally born earlier (median 2006 vs. 2008) and to younger mothers (median age 20 vs. 22). Deceased children were more likely to live in homes with livestock (78% vs. 64%), be born in the long rain season (41% vs. 24%), be part of a multiple-gestation pregnancy (5% vs. 2%), and be born less than 18 months after a previous sibling (29% vs. 11%). Deceased children also seemed less likely to live in homes with electricity, cell phones, and improved pit latrines.

**Table 1:** Descriptive statistics by vital status.

	Child Living (n = 1271)	Child Deceased (n = 91)	Total (n = 1362)
Birth Year	2008 (2004, 2012)	2006 (2002, 2009)	2008 (2004, 2012)
Maternal Age	22 (18, 26)	20 (18, 23)	21 (18, 26)
Birth Order	2 (1, 3)	2 (1, 4)	2 (1, 3)
Age (Years)	9 (5, 13)	-	-
Age at Death (Months)	-	8 (0, 25)	-
Child Gender			
Female	52%	49%	52%
Male	48%	51%	48%
Currently Married/In Relationship			
Yes	93%	97%	93%
No	7%	3%	7%
Respondent's Education			
Secondary or More	21%	18%	21%
Primary	76%	82%	77%
None	3%	0%	3%
Household Electricity			
Yes	13%	8%	13%
No	87%	92%	87%
Household Cell Phone			
Yes	55%	45%	55%
No	45%	55%	45%
Improved Pit Latrine in House			
Yes	13%	7%	13%
No	87%	93%	87%
Household Livestock			
Yes	64%	78%	65%
No	36%	22%	35%
Born During Long Rain Season			
Yes	24%	41%	25%
No	76%	59%	75%
Multiple-Gestation Pregnancy			
Yes	2%	5%	2%
No	98%	95%	98%
Time Since Previous Birth			
≤ 18 months	11%	29%	13%
> 18 months or No Previous	89%	71%	87%

Descriptive statistics by vital status for all children included in the analyses. N = 1,362 for all values except maternal age. N = 1,359 for maternal age due to missing values for 3 children. Categorical variables are presented as percentages, and continuous variables are presented as median (IQR).

### Cause of death

Table 2 shows causes of death among children born alive. The majority of deaths (60%) occurred in the first year of life. The most common causes of death among children under five were malaria, respiratory infection, and anemia, respectively. Respiratory infection was the most common cause of death among infants under one, while malaria was most common among older children. The cause of death was unknown for 22% of children. Cause of death did not change significantly before and after LCA being founded in 2007.

**Table 2:** Cause of death among children born alive.

Cause of Death	Deaths < 1 Year (n = 55)	Deaths 1–5 Years (n = 36)	Total Under-Five Deaths (n = 91)
	n (%)	n (%)	n (%)
Anemia	5 (9%)	5 (14%)	10 (11%)
Congenital Anomalies	1 (2%)	3 (8%)	4 (4%)
Diarrhea	1 (2%)	4 (11%)	5 (5%)
Injury	0 (0%)	1 (3%)	1 (1%)
Malaria	8 (15%)	9 (25%)	17 (19%)
Measles	4 (7%)	3 (8%)	7 (8%)
Prolonged/Complicated Labor	6 (11%)	0 (0%)	6 (7%)
Respiratory Infection	9 (16%)	3 (8%)	12 (13%)
Sickle Cell	3 (5%)	1 (3%)	4 (4%)
Other	4 (7%)	1 (3%)	5 (6%)
Unknown	14 (25%)	6 (17%)	20 (22%)

### Under-five survival analysis

Table 3 shows the results of a multivariable Cox model for U5M. The period between birth and the fifth birthday has been considered together to maximize statistical power. Concordance for the final model was 0.712 (95% CI: 0.661, 0.764).

**Table 3:** Multivariable Cox model for association with under-five mortality.

N = 1,359	Hazard Ratio	95% CI	P-value
Year of Birth	**0.931**	(0.877,0.988)	**0.019**
Maternal Age	0.979	(0.917,1.044)	0.511
Respondent Currently Married/In Relationship	2.619	(0.740,9.269)	0.135
Household has Cell Phone	0.855	(0.519,1.408)	0.539
Improved Pit Latrine in House	0.482	(0.177,1.308)	0.152
Household has Livestock	1.599	(0.877,2.915)	0.125
Born During Long Rain Season	**1.981**	(1.350,2.907)	**<0.001**
Multiple-Gestation Pregnancy	**6.201**	(2.073,18.555)	**0.001**
Time Since Previous Birth > 18 months	**0.345**	(0.203,0.587)	**<0.001**

Hazard ratios for final model of under-five mortality. The multiple pregnancy variable was included as time-varying and turns to zero for all children at the first birthday. All other variables are not time-varying. Bolded text indicates significance at p < 0.05.

The largest risk factor for U5M was multiple-gestation pregnancy (HR 6.201; 95% CI: 2.073, 18.555; p < 0.001). Although only 33 children in the dataset were part of a multiple-gestation pregnancy, 5 died before the fifth birthday (15%). A model containing an indicator for multiple-gestation pregnancy violated the proportional hazards assumption, which is consistent with prior research showing this is a risk factor only in the first year of life [38]. The model presented here contains multiple-gestation pregnancy as a time-varying indicator that changes to zero for all children on the first birthday.

There was a highly significant reduction in mortality with each one year increase in birth year across the observation period (HR 0.931; 95% CI: 0.877, 0.988; p = 0.019). Furthermore, being born in the long rain season (April to June) was associated with an increased likelihood of U5M (HR 1.981; 95% CI: 1.350, 2.907; p < 0.001). A similar, although smaller, effect was seen when analyzing the likelihood of U5M during all rainy seasons compared to dry seasons (HR 1.738; 95% CI: 1.117, 2.705; p = 0.014). This includes the short rain season (October to December) and the long rain season as above. Nearly equal numbers of children were born in the wet and dry period of the year (1,224 vs. 1,382). Being born more than 18 months after the most recent sibling was negatively associated with U5M (HR 0.345; 95% CI: 0.203, 0.587; p < 0.001). Conversely, being born less than 18 months after the closest sibling would translate to an increased likelihood of U5M (HR 2.90).

Living in a household with an improved latrine trended towards being associated with lower U5M (HR 0.482; p = 0.152), while having livestock trended towards an increased likelihood of U5M, though neither reached statistical significance.

### Infant survival analysis

A smaller model of infant (under-one) mortality using only variables that were significant in the U5M model resulted in similar hazard ratios. Infant mortality appeared to decrease more over time than U5M (HR 0.917; 95% CI: 0.870, 0.966; p = 0.001).

### Effect of the Lwala Community Alliance

A time-varying binary indicator that turned to one beginning on January 1, 2007 was created based on the *a priori* hypothesis that child mortality would improve after the establishment of the Lwala Community Alliance and associated hospital. Including this indicator in the regression model from Table 3, in place of birth year, was associated with a decreased likelihood of U5M (HR 0.520; 95% CI: 0.289, 0.936; p = 0.029). Other coefficients and confidence intervals did not change substantially. Similar time-varying indicators beginning in 2005 and 2006 did not yield significant hazard ratios for U5M. Univariate Cox regression in the pre-2007 period yielded a hazard ratio of 1.027 (95% CI: 0.872, 1.210; p = 0.747) for an increased likelihood of U5M for each one year increase in year of birth, while in the post-2007 period this likelihood trended down (HR 0.931; 95% CI: 0.830, 1.044; p = 0.220) for U5M. Similar, though non-significant trends were seen for under-one mortality by each subsequent year of birth over the same time periods.

**Fig 2:**
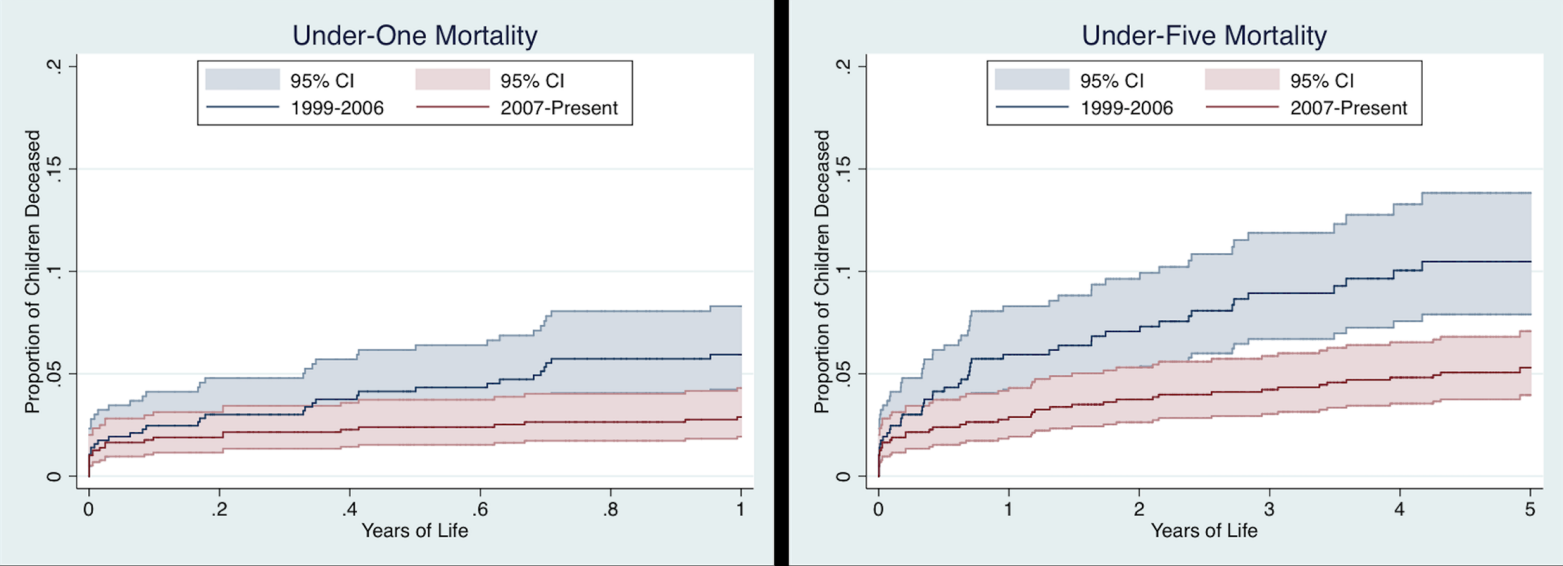
Kaplan-Meier mortality curves. Kaplan-Meier graphs showing failure function for risk experienced before and after January 1^st^, 2007. Shaded areas indicate 95% confidence intervals. The curves are significantly different in both analyses (under-one: p = 0.007; under-five: p < 0.001).

Fig 2 shows Kaplan-Meier curves for before and after January 1, 2007 for both under-one and under-five mortality. These curves are significantly different in both analyses (under-one: p = 0.007; under-five: p < 0.001). The mortality rate in the first year of life was 59.4 per 1,000 live births before 2007 compared to 28.8 per 1,000 live births after 2007. Similarly, the U5M rate was 104.8 per 1,000 live births prior to 2007 and 53.0 per 1,000 afterward.

## Discussion

This dataset captured 1,362 children born since 1999 in a rural Kenyan community near Lake Victoria. 91 of these children died before their fifth birthday. We found malaria (19%), respiratory infections (13%), and anemia (11%) to be the most common causes of U5M in our study population, which is consistent with other studies in rural western Kenya and international studies [3,32,33]. In a similar study in the region, the proportion of mortality due to malaria (28%) and respiratory infections (25%) were higher, as reported by verbal autopsy [32]. This is likely due to the large proportion of cases in our study in which a cause of death was not determined (22%), compared to just 2.1% in the verbal autopsy study. This increased rate of unknown cause may be due to the fact that cause of death was obtained with a single question in place of verbal autopsy. Additionally, all deaths for which cause was unknown occurred in 2012 or earlier. This suggests healthcare access may have improved, and parents may be more likely to receive an explanation of death in more recent years. Alternatively, parents of children may be less likely to remember cause for deaths that occurred longer ago, although this seems less likely considering the importance of such an event. It is also possible that programming by LCA, especially both facility-based and community-based diagnosis and treatment of malaria, may have contributed to this difference in rates of death due to malaria and respiratory infection. These predominant causes suggest that efforts to encourage early and appropriate care seeking by caregivers of children with these conditions may reduce mortality. Determinants of care seeking will be the subject of future research utilizing this dataset to inform implementation efforts.

### Risk factors

Being part of a multiple-gestation pregnancy had the strongest association with U5M in our study. Including multiple-gestation pregnancy in the Cox model violated the proportional hazards assumption, because the effect of this variable is almost entirely within the first year of life. This is consistent with both previous analyses of U5M and studies of perinatal mortality in Kenya [38,43]. Members of multiple-gestation pregnancies accounted for 5.5% of overall under-five deaths in the dataset. Considering the relatively small number of multiple-gestation pregnancies, intense outreach and clinical efforts directed at these children could substantially reduce overall mortality. Importantly, these children are already known to LCA through the Thrive Through Five program and could be found without significant difficulty after discharge from the hospital.

We also found a significant effect of birth during the long rain season on mortality. The effect of birth season on mortality in the literature is somewhat controversial. Although studies have found that child mortality peaks in the rainy season, season of birth has generally not been associated with child mortality beyond the first year of life [38,61,62]. Some studies have supported associations between season of birth and delayed adult mortality through theoretical effects of early childhood malnutrition [56]. Our study finds that the effect of birth season is maintained until the fifth birthday. Although nearly 60% of deaths among children born during the long rain season occurred in the first year of life, a birth-year-adjusted analysis including only the period between the first and fifth birthday still yielded a significant effect of season of birth on mortality (HR 2.53; 95% CI: 1.321, 4.856; p = 0.005). It has been proposed that this increased mortality is secondary to increased malaria exposure and malnutrition secondary to food seasonality [56,63]. This study lends evidence to the fact that these risks extend beyond the first year of life, possibly secondary to delayed effects of neonatal malnutrition [56].

Longer birth spacing has consistently been associated with lower childhood mortality in widely varying contexts [38,57,64–66]. Our study found an effect of similar magnitude with an increased likelihood of mortality (HR 2.90) for children born less than 18 months after a previous sibling. This suggests that family planning and education about birth spacing could substantially reduce child mortality in the region. LCA has extensively focused on providing contraception, including a focus on long-acting reversible methods. Preliminary analyses of family planning questions in this same survey show a contraception utilization rate of 61.5%. This compares favorably to the overall Migori County utilization rate of just 44.6% [6]. Additionally, birth spacing increased after 2007 when LCA was founded (pre-2007 mean 2.2 years; post-2007 3.4 years; p < 0.0001). Additional community outreach could continue to improve both use of contraception and potentially overall child mortality.

Interestingly, we did not find a significant effect of maternal age on U5M. A larger proportion (8%) of children born to mothers under age 18 and an additional 8% of children born to mothers greater than age 35 died before their fifth birthday; compared to only 6% of those born to mothers between 18 and 35 years of age. However, both our final model using maternal age as a continuous variable and a separate Cox regression using categories of age did not show a significant association with mortality. This is likely due to the relatively small number of women giving birth at the extremes of age. Similarly, there is some evidence in the literature that access to improved toilet facilities reduces childhood mortality [67–69]. We did not find a significant association between improved pit latrines and mortality in our study, but living in a household with an improved pit latrine was associated with a trend toward lower likelihood of U5M. This is likely affected by the fact that just 13% of children live in homes that had improved pit latrines, which reduced our ability to detect a statistical difference. Finally, some studies have found an increase in all-cause U5M with livestock ownership, possibly due to infection exposure [44]. However, the magnitude of this effect is small and unlikely to be detected in our relatively small study.

### Effect of the Lwala Community Alliance

A secondary aim of this study was to analyze the possible effect of the presence of the Lwala Community Alliance on child mortality. Mortality curves for both under-one and under-five mortality were significantly different before and after the founding of LCA in 2007. The U5M rate in the pre-2007 era was 104.8 per 1,000 live births compared to 53.0 per 1,000 afterward with similar reductions in under-one mortality. The estimated U5M rate in Nyanza province from 1998–2008 was 149 per 1,000 live births, and from 2004–2014 was 82 per 1,000 [6,70]. At face value, these both appear to be approximately 50% reductions over similar time periods. However, our data suggest that mortality was not dropping in the catchment area in the pre-2007 era with a hazard ratio of 1.027 for each successive year. In contrast, each successive year in the post-2007 period had a hazard ratio of 0.931. This must be interpreted with caution as both hazard ratios were not significantly different from one, but it is corroborated by the fact that being born in the post-LCA period carried a reduced likelihood of mortality (HR 0.520; p = 0.029) compared to the pre-LCA period in an adjusted regression. Sensitivity analyses using breaks in 2005 and 2006 did not yield significant coefficients. Further, the U5M rate continued to drop when examining more recent time periods. For example, among all children captured in the dataset born in the last five years, the U5M rate was 29.5 per 1,000 live births. It is likely that a portion of the mortality reduction during the study period is due to the presence of LCA because of this temporality and the fact that LCA may be providing the majority of formal medical care in the catchment area. Other data in this survey suggest that 90% of respondents have accessed care at LCA for themselves and that nearly 93% have accessed care at LCA for a child.

We did not observe a significant change in cause of death before and after the founding of LCA in 2007. This may be due to the relatively small sample size, which makes changes difficult to interpret. It is also likely that a substantial portion of the reduction in mortality is mediated by very high skilled birth attendant and facility delivery rates. These rates were only available for children under five in the sample and thus could not be included in the model. These rates were 89.7% and 87.9%, respectively. This compares very favorably to the rates for the surrounding county in 2014, which were both 53% [6]. Facility delivery and skilled birth attendants—and the care packages they provide—have reduced maternal and neonatal mortality in many settings [20]. There was a drop in the proportion of deaths due to prolonged or complicated labor from 10% (5 of 48) before 2007 to 2% (1 of 43) after 2007, but the sample is too small to draw definitive conclusions.

### Limitations

The primary limitations to the study are the sample size and the cross-sectional design. Our sample of 1,362 children contained only 91 deaths, which limits the number of variables that can be contained in any one model. Following the rule of 10 events per independent variable [71], we are limited to 9 variables in our overall model. Similarly, we can only include five in any model of under-one mortality because only 55 children died before the first birthday. While this has limited our ability to adjust for some potential confounders, the robustness of significant associations across various models and timespans lends credibility to the findings. For some variables—like improved pit latrines and livestock ownership—this small sample size is compounded by the relative rarity in the sample. This may have limited our ability to detect significance for these variables.

Additionally, the cross-sectional nature of the survey forces us to rely on the memory of respondents about events up to 18 years ago. However, this is likely minimized by the important nature of the birth and death of children. Additionally, this would be expected to bias towards more recent deaths in the dataset, as they are closer to the time of the survey. This was not the case, and there were more deaths recorded earlier in the study period.

The main limitation in our evaluation of LCA is the lack of a direct control group. It is difficult to compare the mortality rates from this study to those found in larger national surveys because both the instruments and the method of calculation differ slightly. This lack of a control group makes it difficult to control for secular trends in mortality rates. However, the fact that mortality dropped by nearly 50% from the pre-2007 period to the post-2007 period in the setting of a constant mortality rate prior to 2007 suggests a large shift around this time period. The only major healthcare change in this rural area during this time period was the presence of LCA, so it is plausible that at least a portion of the reduction in mortality is due to the establishment of this organization and hospital.

## Conclusions

We describe factors associated with childhood mortality—including multiple-gestation pregnancies, short birth intervals, and birth during rainy seasons—in a rural Kenyan community using survival analyses of complete birth histories. This provides both the hyper-local information needed to improve programming and generalizable conclusions for other organizations working in similar environments.

## Supporting information

S1 Appendix Survey tool. The survey was administered on a tablet-based software. Some minor changes from this printed tool were made based on software logistics.
(DOCX)

S2 Appendix Additional model with child gender. An additional model that included child gender did not differ substantially in hazard ratios or significance compared to the model presented in the main body of the manuscript.
(DOCX)

## Abbreviations

CHW: Community Health Worker
HR: Hazard Ratio
IQR: Interquartile Range
LCA: Lwala Community Alliance
MDGs: Millennium Development Goals
SDGs: Sustainable Development Goals
SSA: Sub-Saharan Africa
U5M: Under-Five Mortality
