# Supplementary material for: Under-five mortality in the Rongo Sub-County of Migori County, Kenya: Experience of the Lwala Community Alliance 2007-2017 with evidence from a cross-sectional survey
Source: PLoS One. 2018 Sep 7;13(9):e0203690. doi: 10.1371/journal.pone.0203690 (PMC6128651; doi:10.1371/journal.pone.0203690)
Supplement: S1 Appendix — The survey was administered on a tablet-based software. Some minor changes from this printed tool were made based on software logistics. (DOCX) [file pone.0203690.s001.docx]

**S1 Appendix. Survey tool.**

The survey was administered on a tablet-based software. Some minor changes from this printed tool were made based on software logistics.

**Staff_ID Name of Interviewer**: ____________________________________

**Int_DT Record Day/Month/Year of Survey**: __________________ [Day/Month/Year]

**Res_Area Circle Household’s Area:**

1) Andingo 2) Kadianga 3) Kameji 4) Kuna 5) Lwala 6) Minyenya 7) Ofwanga 8) Sumba 9) Tuk Jowi 10) Uriri

**Selecting the respondent:**

1. Ask how many adults are home (18 years or older).
2. If more than one, randomly select one respondent based on first name letter.
3. Respondent must have lived in North Kamagambo since January 2016, otherwise randomly select the next respondent from the house based on first name letter.
4. Move to a private location where no other adults are present.
5. If no one 18 years or older is present, move to the next household.

Hello. My name is __**[your name]__** and I am working with Lwala Community Alliance located in North Kamagambo. We are conducting a survey about health and other topics all over North Kamagambo.

**[Read the consent form and share with the respondent to read and sign the consent form]**

**START OF SURVEY**

**[Note time of interview start]:** ________________ AM / PM

**Res_1 [Select respondent’s Gender]**

FEMALE **[This is the FEMALE version of the survey]**

**Res_2** In what year you were born?

WRITE YEAR___________

**Res_3** How old will you be **this** year? **[If year of birth and age do not align, reconfirm age and write best estimate]**

WRITE AGE____________

**Dem_Q1** How long have you been living continuously in or near North Kamagambo?

1. ALWAYS → **SKIP to Dem_Q3**
2. 0-1 YEAR
3. 1 YEAR OR LONGER, WRITE NUMBER OF YEARS__________

**Dem_Q2** Before living in North Kamagambo did you previously live in a:

1. CITY
2. TOWN
3. VILLAGE

**Dem_Q3** What is your marital status?

1. NEVER MARRIED
2. MARRIED MONOGOMOUS **[respondent’s husband lives with her only and no other women]**
3. MARRIED POLYGOMOUS

**Dem_Q3a** WRITE # of LIVING CO-WIVES_________ **[respondent’s husband lives with her and at least one other woman]**

1. COHABITATING
2. SEPARATED
3. DIVORCED
4. WIDOWED

**Dem_Q4** What is your religion?

1. NONE
2. CATHOLIC
3. SEVENTH-DAY ADVENTIST
4. PROTESTANT
5. ROHO CHURCH
6. LEGIO MARIA
7. AFRICAN INDEPENDENT CHURCH
8. HINDU
9. ISLAM
10. OTHER, SPECIFY______________________________________________

**Dem_Q5** What is the highest level of school that you completed?

1. NO SCHOOL
2. CLASS 4 OR LESS
3. CLASS 5
4. CLASS 6
5. CLASS 7
6. CLASS 8
7. FORM 1
8. FORM 2
9. FORM 3
10. FORM 4
11. SOME COLLEGE
12. COLLEGE
13. SOME UNIVERSITY
14. UNIVERSITY
15. POST-GRADUATE

**HSHLD_Q1** Now I am going to ask you about your family. Have you ever given birth?

1. YES
2. NO **→** **SKIP to HSHLD_Q11**

**HSHLD_Q2** How many **total** pregnancies have you had that resulted in a **live birth**, including sons and daughters who were born alive but later died?

WRITE NUMBER________

**HSHLD_Q3** How many of those sons and daughters are now living with you in your house? **[Including those away at boarding school]**

WRITE NUMBER________

**HSHLD_Q4** How many sons and daughters are alive but do not live with you? **[Excluding those away at boarding school]**

WRITE NUMBER________

[App checks for errors]

**HSHLD_Q5** How many children have you ever given birth to who were born alive but later died?

1. NONE **→** **SKIP to HSHLD_Q8**WRITE NUMBER________

**HSHLD_Q6** How many boys have died?

WRITE NUMBER________

**HSHLD_Q7** How many girls have died?

WRITE NUMBER________

**HSHLD_Q8** How many pregnancies have you had that did not result in a live birth? **[That is, the baby was not breathing, moving, or showing signs of life upon delivery]**

WRITE NUMBER________

**HSHLD_Q9** Since 2011, have any of your children died before reaching their 5th birthday? **[That is, children who have died since *January 2011*, and who died before they reached their *5th birthday*]**

1. YES
2. NO

**HSHLD_Q10** For each pregnancy that resulted in a live birth, how many children are 18 or younger, *including those children that were born alive and later died before their 19th birthday*? **[Please ensure that the respondent includes the number of children who were born alive and later died (#)]**

WRITE NUMBER________

[App checks for errors]

**HSHLD_Q11** Outside of any children you may have delivered, how many 18 or younger children do you have that usually stay in the home or at boarding school?

1. NONE **→** **SKIP to FHSHLD_Q14**WRITE NUMBER________

**HSHLD_Q12** For each of these children, please tell me some more information. **[Include twins separately]**

**Confirm that *(#)* is the total number of 18 or younger children who were born alive, including the children who later died, and any other children that stay in the respondents home. If this number is incorrect, please revise the last two questions.**

| **What name was given to your (oldest/next) child (or child living with you)?** | **What is the respondent’s relationship to (NAME)?** | **Is (NAME) a boy or a girl?** | **On what day, month, and year was (NAME) born?** | **Is (NAME) still alive?** | **IF ALIVE:**  **How old was (NAME) at (NAME)’s last birthday? (RECORD in days if less than 1 month, months if less than two years, or years)** | **IF DEAD:**  **How old was (NAME) when he/she died?**  **(RECORD in days if less than 1 month, months if less than two years, or years)** | **Date of child’s death** | **CAUSE OF DEATH** |
| --- | --- | --- | --- | --- | --- | --- | --- | --- |
| *Ex. Vincent Okoth* | *MOTHER* | *Male* | *11 October 2011* | *Yes or No* | *Age in years (3)* | *YEARS 3* | *24 October 2014* | *Malaria / Anemia* |
|  |  |  |  |  |  | DAYS ______  MONTHS ______  YEARS ______ |  |  |
|  |  |  |  |  |  | DAYS ______  MONTHS ______  YEARS ______ |  |  |
|  |  |  |  |  |  | DAYS ______  MONTHS ______  YEARS ______ |  |  |
|  |  |  |  |  |  | DAYS ______  MONTHS ______  YEARS ______ |  |  |
|  |  |  |  |  |  | DAYS ______  MONTHS ______  YEARS ______ |  |  |
|  |  |  |  |  |  | DAYS ______  MONTHS ______  YEARS ______ |  |  |
|  |  |  |  |  |  | DAYS ______  MONTHS ______  YEARS ______ |  |  |
|  |  |  |  |  |  | DAYS ______  MONTHS ______  YEARS ______ |  |  |
|  |  |  |  |  |  | DAYS ______  MONTHS ______  YEARS ______ |  |  |
|  |  |  |  |  |  | DAYS ______  MONTHS ______  YEARS ______ |  |  |
|  |  |  |  |  |  | DAYS ______  MONTHS ______  YEARS ______ |  |  |
|  |  |  |  |  |  | DAYS ______  MONTHS ______  YEARS ______ |  |  |
|  |  |  |  |  |  | DAYS ______  MONTHS ______  YEARS ______ |  |  |

**HSHLD_Q13** For children who are 5-18:

| **CURRENTLY IN SCHOOL**  (Yes/No) | **TYPE OF SCHOOL ATTENDED** (Gov’t/Private) | **MOST RECENTLY COMPLETED CLASS** (for Class 8 or Form 4 this includes passing exams) |
| --- | --- | --- |
| *No* | *Gov’t* | *Class 7* |
|  |  |  |
|  |  |  |
|  |  |  |
|  |  |  |
|  |  |  |
|  |  |  |
|  |  |  |
|  |  |  |
|  |  |  |
|  |  |  |
|  |  |  |

**HSHLD_Q14** Now tell me the total number of *males*, including children and adults, living in your house right now, including any children you’ve already told me about. **[Make sure respondent counts boys at boarding school]**

WRITE NUMBER OF MALES__________

**HSHLD_Q15** Now tell me the total number of *females*, including children and adults, living in your house right now, including any children you’ve already counted. **[Make sure respondent counts herself and girls at boarding school]**

WRITE NUMBER OF FEMALES_________

**HSHLD_Q16** Are you currently pregnant?

1. YES
2. NO **→** **SKIP to FP_Q1**

**HSHLD_Q17** How many months pregnant are you?

WRITE NUMBER OF MONTHS_________

**FP_Q1 [If respondent is over the age of 50, SKIP → FP_Q6; if respondent is pregnant, SKIP → FP_Q6, otherwise CONTINUE]**

Remember, the information I ask will be kept confidential. Have you or your spouse *ever* used any method of family planning, that is contraception, or done something to delay or avoid getting pregnant?

1. YES
2. NO **→** **SKIP to FP_Q6**
3. DON’T KNOW **→** **SKIP to FP_Q6**

**FP_Q2** Are you or your spouse *currently* doing something or using any method of family planning to delay or avoid getting pregnant?

1. YES
2. NO **→** **SKIP to FP_Q6**
3. DON’T KNOW **→** **SKIP to FP_Q6**

**FP_Q3** Which method(s) are you or your spouse currently using to avoid getting pregnant? **[Do not prompt] [Select All that Apply]**

1. IUD / IUCD / COIL
2. INJECTABLES (SUCH AS *DEPO*)
3. IMPLANTS (SUCH AS *IMPLANON* OR *JADELLE*)
4. PILL
5. CONDOM
6. FEMALE CONDOM
7. FEMALE STERILIZATION
8. MALE STERILIZATION
9. DIAPHRAGM
10. FOAM/JELLY
11. LACTATIONAL AMEN, METHOD
12. RHYTHM METHOD
13. WITHDRAWAL
14. OTHER MODERN METHOD
15. OTHER TRADITIONAL METHOD
16. DON’T KNOW

**FP_Q4 Only ask if 1, 2, 3, 4, or 5 were selected on previous question, otherwise → SKIP to FP_Q6]** When was this method of family planning given to you/your spouse, most recently?

1. 0 - 6 MONTHS AGO
2. 7 - 12 MONTHS AGO
3. 1 - 2 YEARS AGO
4. 2 - 3 YEARS AGO
5. 3 - 4 YEARS AGO
6. MORE THAN 4 YEARS AGO

99. DON’T KNOW

**FP_Q5** Where did you/spouse receive this method of family planning the last time you got it?

1. LWALA COMMUNITY HOSPITAL
2. MINYENYA DISPENSARY
3. NDEGE ORIEDO DISPENSARY
4. RONGO DISTRICT HOSPITAL
5. ROYAL HOSPITAL RONGO
6. OTHER, SPECIFY_____________________________________________________
7. DON’T KNOW

**FP_Q6 [If respondent is married, CONTINUE, otherwise, → SKIP to FP_Q7]**

In the last year, how often did you talk with your spouse about family planning or contraception?

1. NEVER
2. ONCE OR TWICE
3. THREE OR MORE TIMES

**FP_Q7** Again, I won’t share any of the information you give me. Would you say you approve or disapprove of couples using a family planning method to avoid pregnancy?

1. DISAPPROVE
2. APPROVE
3. NO OPINION
4. DON’T KNOW

**FP_Q8 [If respondent has had at least one child, CONTINUE, otherwise, → SKIP to HIV_Q1]** How old were you when you gave birth to your first child?

WRITE WOMAN’S AGE IN YEARS___________

**FP_Q9** When you got pregnant with your last child, that is the last born or your current pregnancy, did you plan to get pregnant at that time?

1. YES **→** **SKIP to HIV_Q1**
2. NO, DID NOT PLAN TO GET PREGNANT

**FP_Q10** At that time, did you want to wait to have that baby until later, or did you want no more children?

1. WANTED TO WAIT LONGER
2. WANTED NO MORE CHILDREN **→** **SKIP to FP_Q12**

**FP_Q11** How much longer did you want to wait?

1. 1-12 MONTHS
2. 12-24 MONTHS
3. 2-3 YEARS
4. 3-4 YEARS
5. 4-5 YEARS
6. MORE THAN 5 YEARS
7. DON’T KNOW

**FP_Q12** Were you able to access contraception or family planning at the time that you conceived your last baby?

1. YES
2. NO

**HIV_Q1** Now I would like to talk about something else. As a reminder, all of your answers will be kept confidential. Have you ever heard of an illness called HIV/AIDS?

1. YES
2. NO **→** **SKIP to WASH_Q1**

I want to assure you that I do not want to know and will not ask about the results of any HIV/AIDS tests you or your spouse or family has received. I am going to read a series of statements about HIV/AIDS. Please answer whether you strongly disagree, disagree, agree, or strongly agree with each statement.

**HIV_Q2** Some people think that those with HIV are disgusting

1. Strongly disagree
2. Disagree
3. Agree
4. Strongly agree

**HIV_Q3** Some people do not want those with HIV playing with their children

1. Strongly disagree
2. Disagree
3. Agree
4. Strongly agree

**HIV_Q4** Some people feel uncomfortable being near those with HIV

1. Strongly disagree
2. Disagree
3. Agree
4. Strongly agree

**HIV_Q5** Some people do not want to talk to others with HIV

1. Strongly disagree
2. Disagree
3. Agree
4. Strongly agree

**HIV_Q6** Some people keep distance from people with HIV

1. Strongly disagree
2. Disagree
3. Agree
4. Strongly agree

**HIV_Q7** Some people think God is punishing people with HIV

1. Strongly disagree
2. Disagree
3. Agree
4. Strongly agree

**HIV_Q8** Some people think demons are punishing people with HIV

1. Strongly disagree
2. Disagree
3. Agree
4. Strongly agree

**HIV_Q9** If a person has HIV, some community members will behave differently towards that person for the rest of his or her life

1. Strongly disagree
2. Disagree
3. Agree
4. Strongly agree

**HIV_Q10** Some people try not to touch others with HIV

1. Strongly disagree
2. Disagree
3. Agree
4. Strongly agree

**HIV_Q11** Some people are afraid of those with HIV

1. Strongly disagree
2. Disagree
3. Agree
4. Strongly agree

**HIV_Q12** Some people think that people with HIV are unclean

1. Strongly disagree
2. Disagree
3. Agree
4. Strongly agree

**HIV_Q13** Some people prefer not to have those with HIV living in their community

1. Strongly disagree
2. Disagree
3. Agree
4. Strongly agree

**HIV_Q14** Some people think that people with HIV get what they deserve

1. Strongly disagree
2. Disagree
3. Agree
4. Strongly agree

**HIV_Q15** Some people who have HIV refuse to believe that they have HIV

1. Strongly disagree
2. Disagree
3. Agree
4. Strongly agree

**HIV_Q16** Some people who have HIV feel hurt because of how others react to knowing they have HIV

1. Strongly disagree
2. Disagree
3. Agree
4. Strongly agree

**HIV_Q17** Some people who have HIV feel alone

1. Strongly disagree
2. Disagree
3. Agree
4. Strongly agree

**HIV_Q18** Some people who have HIV are afraid that other people in the community will talk about them having HIV

1. Strongly disagree
2. Disagree
3. Agree
4. Strongly agree

**HIV_Q19** Some people who have HIV lose friends when they share with them they have HIV

1. Strongly disagree
2. Disagree
3. Agree
4. Strongly agree

**HIV_Q20** Some people who have HIV lose family support when they share with them they have HIV

1. Strongly disagree
2. Disagree
3. Agree
4. Strongly agree

**HIV_Q21** Some people who have HIV are afraid to tell their spouse that they have HIV

1. Strongly disagree
2. Disagree
3. Agree
4. Strongly agree

**HIV_Q22** Some people who have HIV are afraid to tell those outside their family that they have HIV

1. Strongly disagree
2. Disagree
3. Agree
4. Strongly agree

**HIV_Q23** Some people know they have HIV but are afraid to go to the clinic to get medicine for HIV

1. Strongly disagree
2. Disagree
3. Agree
4. Strongly agree

**HIV_Q24** Some people who have HIV worry that others will reveal their secret

1. Strongly disagree
2. Disagree
3. Agree
4. Strongly agree

**HIV_Q25** Some people who have HIV try very hard to keep the issue of having HIV a secret

1. Strongly disagree
2. Disagree
3. Agree
4. Strongly agree

**HIV_Q26** Some people who have HIV keep their distance from others to avoid spreading the HIV virus

1. Strongly disagree
2. Disagree
3. Agree
4. Strongly agree

**HIV_Q27** Some people who have HIV feel guilty because their family has the burden of caring for them

1. Strongly disagree
2. Disagree
3. Agree
4. Strongly agree

**HIV_Q28** Some people who have HIV will choose carefully who they tell about having HIV

1. Strongly disagree
2. Disagree
3. Agree
4. Strongly agree

**HIV_Q29** In your opinion, what do you think keeps community members from enrolling in HIV care? **[Do not prompt] [Select all that apply]**

1. DENIAL / FEAR OF ACCEPTING STATUS
2. FEAR OF ISOLATION / STIGMA
3. RELIGIOUS BELIEFS
4. FEAR OF LOSING THE SUPPORT OF A SPOUSE / BEING REJECTED BY A SPOUSE
5. FEAR OF VIOLENCE FROM SPOUSE
6. FEAR OF LOSING THE SUPPORT OF / BEING REJECTED BY FRIENDS OR RELATIVES
7. FEAR OF OTHER COMMUNITY MEMBERS FINDING OUT
8. FEAR OF EMPLOYER FINDING OUT
9. FEAR OF GOING TO A HEALTH FACILITY FOR HIV MEDICINE
10. CONCERNS ABOUT CONFIDENTIALITY AMONG HEALTH CARE WORKERS OR FACILITY STAFF
11. OTHER, SPECIFY ___________________________________________________

**HIV_Q30** I don't want to know the results, but have you ever been tested for HIV/the AIDS virus?

1. YES **→** **SKIP to question HIV_Q32**
2. NO
3. DON’T KNOW

**HIV_Q31** What is keeping you from taking an HIV test? **[Select all that apply]**

- 1. DENIAL / FEAR OF ACCEPTING STATUS **→** **SKIP to LCA_Q1**
  2. FEAR OF ISOLATION / STIGMA **→** **SKIP to LCA_Q1**
  3. RELIGIOUS BELIEFS **→** **SKIP to LCA_Q1**
  4. FEAR OF TAKING HIV MEDICINE **→** **SKIP to LCA_Q1**
  5. FEAR OF A SPOUSE FINDING OUT **→** **SKIP to LCA_Q1**
  6. FEAR OF FRIENDS OR RELATIVES FINDING OUT **→** **SKIP to LCA_Q1**
  7. FEAR OF OTHER COMMUNITY MEMBERS FINDING OUT **→** **SKIP to LCA_Q1**
  8. FEAR OF EMPLOYER FINDING OUT **→** **SKIP to LCA_Q1**
  9. CONCERNS ABOUT CONFIDENTIALITY AMONG HEALTH CARE WORKERS OR FACILITY STAFF **→** **SKIP to LCA_Q1**
  10. COST OF TEST / TREATMENT **→** **SKIP to LCA_Q1**
  11. I DON’T KNOW WHERE TO GET AN HIV TEST **→** **SKIP to LCA_Q1**

1. OTHER, SPECIFY__________________________________ **→** **SKIP to LCA_Q1**

**HIV_Q32** Where have you been tested? **[Select all that apply]**

1. HEALTH FACILITY WHEN I WAS PREGNANT
2. HEALTH FACILITY WHEN I DELIVERED
3. HEALTH FACILITY WHEN I WAS TREATED AS AN OUTPATIENT
4. HEALTH FACILITY WHEN WAS ADMITTED AS AN INPATIENT
5. HEALTH FACILITY WHEN I WENT FOR THE TEST
6. AT AN OUTREACH EVENT IN THE COMMUNITY
7. AT A VOLUNTARY COUNSELING AND TESTING CENTER
8. OTHER, SPECIFY__________________________________________________

**HIV_Q33** How many months ago was your most recent HIV test?

1. 1 – 6 MONTHS
2. 6 - 12 MONTHS
3. 12-24 MONTHS
4. TWO OR MORE YEARS
5. DON’T KNOW

**HIV_Q34** Did you share the results of the test with your husband or partner(s)?

1. YES
2. NEVER RECEIVED RESULTS
3. NO
4. NOT APPLICABLE, NO HUSBAND / PARTNER

**WASH_Q1** Now we are going to talk about something else. Currently, what is the main source for drinking water for members in your household? **[do not prompt]**

1. TUBE WELL OR BOREHOLE
2. DUG WELL-PROTECTED WELL
3. DUG WELL-UNPROTECTED WELL
4. WATER FROM SPRING-PROTECTED SPRING
5. WATER FROM SPRING-UNPROTECTED SPRING
6. RAINWATER
7. TANKER TRUCK
8. CART WITH SMALL TANK
9. SURFACE WATER (RIVER/POND/STREAM/IRRIGATION CHANNEL)
10. BOTTLED WATER
11. PIPED WATER- PIPED INTO DWELLING
12. PIPED WATER- PIPED TO YARD/PLOT
13. PIPED WATER- PIPED TO NEIGHBOR
14. PIPED WATER- PUBLIC TAP/STANDPIPE
15. OTHER, SPECIFY___________________________________________

**WASH_Q2** Currently, what is the main source of water used by your household for other purposes such as cooking or washing? **[do not prompt]**

1. TUBE WELL OR BOREHOLE
2. DUG WELL-PROTECTED WELL
3. DUG WELL-UNPROTECTED WELL
4. WATER FROM SPRING-PROTECTED SPRING
5. WATER FROM SPRING-UNPROTECTED SPRING
6. RAINWATER
7. TANKER TRUCK
8. CART WITH SMALL TANK
9. SURFACE WATER (RIVER/POND/STREAM/IRRIGATION CHANNEL)
10. BOTTLED WATER
11. PIPED WATER- PIPED INTO DWELLING
12. PIPED WATER- PIPED TO YARD/PLOT
13. PIPED WATER- PIPED TO NEIGHBOR
14. PIPED WATER- PUBLIC TAP/STANDPIPE
15. OTHER, SPECIFY_______________________________________________

**WASH_Q3** Do you usually do something to the water to make it safer to drink?

1. YES
2. NO **→** **SKIP to WASH_Q5**
3. DON’T KNOW **→** **SKIP to WASH_Q5**

**WASH_Q4** What do you usually do to make the water safer to drink? **[Select all that apply]**

1. BOIL
2. ADD BLEACH/CHLORINE AT HOME (SUCH AS WATERGUARD OR AQUATAB)
3. ADD CHLORINE FROM BLUE DISPENSER AT SOURCE
4. USE WATER FILTER (SUCH AS CERAMIC/SAND/COMPOSITE)
5. SOLAR DISINFECTION
6. LET IT STAND AND SETTLE
7. OTHER, SPECIFY______________________________________________
8. DON’T KNOW

**WASH_Q5** Have you or anyone in this household, 18 years or older, received an official training in WASH, that is in water, sanitation and hygiene? **[Explain as necessary that a training is in proper hygiene, clean water, latrine construction] [Select all that apply]**

1. YES, 1 DAY TRAINING OR LESS
2. YES, 2 OR 3 DAY TRAINING
3. YES, 4 DAY TRAINING
4. YES, OTHER, SPECIFY_________________________________________
5. NO **→** **SKIP to WASH_Q7**
6. DON’T KNOW **→** **SKIP to WASH_Q7**

**WASH_Q6** Who was responsible for the WASH training that you or other household members received? **[Select all that apply]**

1. LWALA COMMUNITY ALLIANCE
2. ANOTHER NON-GOVERNMENT/PRIVATE ORGANIZATION
3. GOVERNMENT/MINISTRY OF HEALTH OR PUBLIC HEALTH

88. OTHER, SPECIFY____________________________________________

99. DON’T KNOW

**WASH_Q7** Has your household ever been visited by a Community Health Worker (CHW) or Umama Salama?

1. YES
2. NO **→** **SKIP to WASH_Q12**
3. DON’T KNOW **→** **SKIP to WASH_ Q12**
4. DON’T KNOW WHAT A CHW OR UMAMA SALAMA IS → **SKIP to WASH_ Q12**

**WASH_Q8** In the past 3 months, how many times has a Community Health Worker (CHW) or Umama Salama from Lwala Community Alliance visited your household?

1. 0 VISITS **→** **SKIP to WASH_ Q12**
2. 1 VISIT
3. 2 VISITS
4. 3 VISITS
5. MORE THAN 3 VISITS
6. DON’T KNOW

**WASH_Q9** In the past 1 month, how many times has a Community Health Worker (CHW) or Umama Salama from Lwala Community Alliance visited your household?

1. 0 VISITS
2. 1 VISIT
3. 2 VISITS
4. 3 VISITS
5. MORE THAN 3 VISITS
6. DON’T KNOW

**WASH_Q10** What is the name of the CHW or Umama Salama who visits you most frequently?

1. RESPONDENT CANNOT RECALL CHW NAME
2. WRITE CHW FULL NAME: _________________________________

**WASH_Q11** During the most recent visit, what did the CHW or Umama Salama from Lwala Community Alliance do when they visited your household? **[Select all that apply]**

1. PROVIDE REFERRAL TO HEALTH FACILITY
2. CHECK ON CHILD’S IMMUNIZATION STATUS
3. CHECK ON PREGNANCY STATUS/PRENATAL CARE VISIT ATTENDANCE
4. ANSWER HEALTH QUESTIONS
5. PROVIDE FAMILY PLANNING COUNSELING
6. PROVIDE EMOTIONAL OR SOCIAL SUPPORT
7. ASSESS HEALTH OF CHILDREN
8. PROVIDE HEALTH EDUCATION (SUCH AS HIV, MALARIA, DIARRHEA, ANC)
9. TALK ABOUT LATRINES OR OTHER WASH TOPICS
10. TALK ABOUT MEDICATIONS OR TREATMENT
11. HOME-BASED DIAGNOSIS AND TREATMENT
12. COLLECT INFORMATION ABOUT YOU OR YOUR HOUSEHOLD
13. PROVIDE WATER PURIFICATION TABLETS
14. OTHER, SPECIFY__________________________________________________
15. DON’T KNOW

**WASH_Q12** How often would you like a CHW to visit your household in the next 1 month?

1. 1 TIME
2. 2 TIMES
3. 3 TIMES
4. 4 OR MORE TIMES
5. NEVER
6. DON’T KNOW OR NO OPINION

**WASH_Q13** What type of services would you like to receive at your home?

1. PROVIDE REFERRAL TO HEALTH FACILITY
2. CHECK ON CHILD’S IMMUNIZATION STATUS
3. CHECK ON PREGNANCY STATUS/PRENATAL CARE VISIT ATTENDANCE
4. ANSWER HEALTH QUESTIONS
5. PROVIDE FAMILY PLANNING COUNSELING
6. PROVIDE EMOTIONAL OR SOCIAL SUPPORT
7. ASSESS HEALTH OF CHILDREN
8. PROVIDE HEALTH EDUCATION (SUCH AS HIV, MALARIA, DIARRHEA, ANC)
9. TALK ABOUT LATRINES OR OTHER WASH TOPICS
10. TALK ABOUT MEDICATIONS OR TREATMENT
11. HOME-BASED DIAGNOSIS AND TREATMENT
12. COLLECT INFORMATION ABOUT YOU OR YOUR HOUSEHOLD
13. PROVIDE WATER PURIFICATION TABLETS
14. OTHER, SPECIFY______________________________________________
15. DON’T KNOW

**[If respondent has given birth to at least living child under the age of five, CONTINUE, otherwise, → SKIP to MENT_Q1]**

**CHH_Q1** Now I want to know about the health of your youngest child under 5 years of age who is alive. Where did you give birth to **[child’s name]**?

1. YOUR HOME
2. OTHER HOME
3. LWALA COMMUNITY ALLIANCE HOSPITAL
4. OTHER PRIVATE HOSPITAL/CLINIC
5. GOVERNMENT HEALTH DISPENSARY, *MINYENYA* OR *NDEGE ORIEDO*
6. GOVERNMENT HEALTH DISPENSARY, *OTHER*
7. GOVERNMENT HOSPITAL
8. OTHER, SPECIFY_____________________________________________
9. DON’T KNOW

**CHH_Q2** Who assisted with the delivery of **[child’s name]**?

1. NO ONE ASSISTED
2. CLINICIAN OR NURSE
3. DOCTOR
4. TRAINED OR CERTIFIED MIDWIFE
5. TRADITIONAL BIRTH ATTENDANT
6. RELATIVE/FRIEND
7. OTHER, SPECIFY______________________________________________

**CHH_Q3** During the pregnancy of **[child’s name]** did you receive an injection in the arm to prevent the baby from getting tetanus, that is, convulsions after birth?

1. YES
2. NO **→** **SKIP to CHH_Q5**
3. DON’T KNOW **→** **SKIP to CHH_Q5**

**CHH_Q4** How many times did you receive such an injection?

1. ONE TIME
2. TWO TIMES
3. MORE THAN TWO TIMES
4. DON’T KNOW

**CHH_Q5** During your pregnancy with **[child’s name]**, how many antenatal care (ANC) visits did you attend at a health facility before you delivered**?**

1. 0 VISITS **→** **SKIP to CHH_Q7**
2. 1 VISIT
3. 2 VISITS
4. 3 VISITS
5. 4 VISITS
6. 5 VISITS
7. 6 OR MORE VISITS
8. DON’T KNOW

**CHH_Q6** Did your husband/partner attend any of the ANC visits with you?

1. YES
2. NO
3. DON’T KNOW

**CHH_Q7** Now I would like to ask you about the types of liquids and foods that **[child’s name]** consumed yesterday during the day or at night. Since yesterday morning, did **[child’s name]** take… **[Read each of the following and mark each item consumed by the child]**

1. BREASTMILK
2. PLAIN WATER
3. MILK
4. PORRIDGE
5. SODA
6. OTHER LIQUIDS
7. CANDY OR SWEETS
8. VEGETABLES (SUCH AS SIKUMA OR TOMATO)
9. FRUIT (SUCH AS AVACADO OR MANGO)
10. JUGGARY
11. UGALI
12. RICE OR POTATO
13. BEANS
14. EGGS
15. MEAT (SUCH AS FISH, BEEF, LIVER, OR CHICKEN)
16. DON’T KNOW

**CHH_Q8** In the last two weeks, has **[child’s name]** been ill with a fever at any time?

1. YES
2. NO **→** **SKIP to CHH_Q18**
3. DON’T KNOW **→** **SKIP to CHH_Q18**

**CHH_Q9** Did you seek advice or treatment for the fever? **[Including medicine from a chemist]**

1. YES
2. NO **→** **SKIP to CHH_Q18**
3. DON’T KNOW **→** **SKIP to CHH_Q18**

**CHH_Q10** Where did you seek advice or treatment? **[Do not prompt]** **[Select all that apply]**

1. LWALA COMMUNITY ALLIANCE HOSPITAL
2. OTHER PRIVATE HOSPITAL/CLINIC
3. GOVERNMENT HEALTH DISPENSARY, *MINYENYA* OR *NDEGE ORIEDO*
4. GOVERNMENT HEALTH DISPENSARY, *OTHER*
5. GOVERNMENT HOSPITAL
6. MOBILE CLINIC/ CLINICAL OUTREACH
7. COMMUNITY HEALTH WORKER
8. TRADITIONAL HEALER
9. FAMILY MEMBER
10. CHEMIST
11. OTHER, SPECIFY____________________________________________________
12. DON’T KNOW

**CHH_Q11** When you sought advice or treatment for the fever, was a malaria rapid diagnostic test (RDT) done to **[child’s name]**?

1. YES **→** **SKIP to CHH_Q13**
2. NO
3. DON’T KNOW

**CHH_Q12** At that time, was a laboratory test (blood smear) for malaria done to **[child’s name]**?

1. YES
2. NO **→** **SKIP to CHH_Q15**
3. DON’T KNOW **→** **SKIP to CHH_Q15**

**CHH_Q13** Was the result (RDT/Blood smear) positive for malaria?

1. YES
2. NO **→** **SKIP to CHH_Q15**
3. DON’T KNOW **→** **SKIP to CHH_Q15**

**CHH_Q14** If the RDT/Blood smear was positive, what was the treatment given to **[child’s name]**?

1. COARTEM (AL) CP **→** **SKIP to CHH_Q17**
2. AMODIAQUINA + ARTESUNATO (ASAQ) **→** **SKIP to CHH_Q17**
3. FANSIDAR CP **→** **SKIP to CHH_Q17**
4. QUININO CP **→** **SKIP to CHH_Q17**
5. QUININO INJ **→** **SKIP to CHH_Q17**
6. ARTESUNATO **→** **SKIP to CHH_Q17**
7. PARACETAMOL COMPRIMIDO/XAROPE **→** **SKIP to CHH_Q17**
8. OTHER, SPECIFY____________________________________________________ **→** **SKIP to CHH_Q17**
9. DON’T KNOW **→** **SKIP to CHH_Q17**

**CHH_Q15**If the RDT/Blood smear was negative or if [child’s name] was never tested, was [child’s name] treated?

1. YES
2. NO **→** **SKIP to CHH_Q18**
3. DON’T KNOW **→** **SKIP to CHH_Q18**

**CHH_Q16**What was the treatment given to [child’s name]?

1. COARTEM (AL) CP
2. AMODIAQUINA + ARTESUNATO (ASAQ)
3. FANSIDAR CP
4. QUININO CP
5. QUININO INJ
6. ARTESUNATO
7. PARACETAMOL COMPRIMIDO/XAROPE
8. OTHER, SPECIFY____________________________________________________
9. DON’T KNOW

**CHH_Q17** Did **[child’s name]** take the antimalarial treatment the same day or the day after the onset of the fever?

1. YES
2. NO
3. DON’T KNOW

**CHH_Q18** In the last two weeks, has **[child’s name]** had diarrhea at any time?

1. YES
2. NO **→** **SKIP to CHH_Q22**
3. DON’T KNOW **→** **SKIP to CHH_Q22**

**CHH_Q19** Did you seek advice or treatment for the diarrhea?

1. YES
2. NO **→** **SKIP to CHH_Q22**
3. DON’T KNOW **→** **SKIP to CHH_Q22**

**CHH_Q20** Where did you seek advice or treatment? **[Do not prompt]** **[Select all that apply]**

1. LWALA COMMUNITY ALLIANCE HOSPITAL
2. OTHER PRIVATE HOSPITAL/CLINIC
3. GOVERNMENT HEALTH DISPENSARY, *MINYENYA* OR *NDEGE ORIEDO*
4. GOVERNMENT HEALTH DISPENSARY, *OTHER*
5. GOVERNMENT HOSPITAL
6. MOBILE CLINIC/ CLINICAL OUTREACH
7. COMMUNITY HEALTH WORKER
8. TRADITIONAL HEALER
9. FAMILY MEMBER
10. CHEMIST
11. OTHER, SPECIFY____________________________________________________
12. DON’T KNOW

**CHH_Q21** What was the treatment you used for this diarrhea? **[Select all that apply]**

1. NO TREATMENT GIVEN
2. MORE FOOD
3. MORE WATER
4. ORAL REHYDRATION THERAPY (ORS)
5. MEDICINE/DRUGS (SUCH AS FLAGGYL ANTIBIOTIC)
6. TRADITIONAL REMEDY
7. OTHER, SPECIFY_____________________________________________________
8. DON’T KNOW

**CHH_Q22** In the last two weeks, has **[child’s name]** had a cough or difficult or shallow breathing?

1. YES → **SKIP to CHH_Q24**
2. NO
3. DON’T KNOW

**CHH_Q23** In the last 12 months, has **[child’s name]** had a cough or difficult or shallow breathing?

1. YES
2. NO → **SKIP to CHH_Q26**
3. DON’T KNOW → **SKIP to CHH_Q26**

**CHH_Q24** Did you seek advice or treatment for the breathing problem?

1. YES
2. NO → **SKIP to CHH_Q29**
3. DON’T KNOW → **SKIP to CHH_Q29**

**CHH_Q25** Where did you seek advice or treatment for the breathing problem? **[Do not prompt] [Select all that apply]**

1. LWALA COMMUNITY ALLIANCE HOSPITAL
2. OTHER PRIVATE HOSPITAL/CLINIC
3. GOVERNMENT HEALTH DISPENSARY, *MINYENYA* OR *NDEGE ORIED*
4. GOVERNMENT HEALTH DISPENSARY, *OTHER*
5. GOVERNMENT HOSPITAL
6. MOBILE CLINIC/ CLINICAL OUTREACH
7. COMMUNITY HEALTH WORKER
8. TRADITIONAL HEALER
9. FAMILY MEMBER
10. CHEMIST
11. OTHER, SPECIFY____________________________________________________
12. DON’T KNOW

**CHH_Q26** Can you tell me what symptoms indicate that a child needs to be taken to a health facility? Please tell me all the symptoms that you can. **[Do not prompt] [Select all that apply]**

1. NO ANSWER GIVEN
2. LOOKS UNWELL OR NOT PLAYING NORMALLY
3. FEVER OR CHILLS
4. NOT EATING OR DRINKING
5. VOMITS EVERYTHING
6. LETHARGIC OR DIFFICULT TO WAKE
7. PERSISTENT COUGH
8. FAST OR DIFFICULT BREATHING
9. DIARRHEA
10. BLOODY STOOL
11. CONVULSIONS
12. OTHER, SPECIFY_____________________________________________________

**CHH_Q27** In your understanding, tell me some of the ways that you can identify a child who is malnourished? **[Do not prompt]** **[Select all that apply]**

1. NO ANSWER GIVEN
2. SUNKEN EYES
3. DECREASING BODY WEIGHT
4. DISCOLORED HAIR
5. SMALL BODY SIZE
6. THIN LIMBS
7. EXTENDED BELLY
8. FATIGUE/LETHARGY
9. FLABBY CHEEKS
10. PALE SKIN
11. OTHER, SPECIFY___________________________________________________

**Now I am going to ask you some questions about your own health.**

**MENT_Q1** In the last two weeks, how many days did you have little interest or little happiness in doing things?

1. NO DAYS
2. LESS THAN 1 WEEK
3. 1 WEEK OR MORE
4. ALMOST ALL DAYS
5. DON’T KNOW
6. PREFER NOT TO ANSWER

**MENT_Q2** In the last two weeks, how many days did you feel down, depressed or without motivation?

1. NO DAYS
2. LESS THAN 1 WEEK
3. 1 WEEK OR MORE
4. ALMOST ALL DAYS
5. DON’T KNOW
6. PREFER NOT TO ANSWER

**MENT_Q3** In the last two weeks, how many days did you have difficulty sleeping, staying asleep, or sleeping more than is customary?

1. NO DAYS
2. LESS THAN 1 WEEK
3. 1 WEEK OR MORE
4. ALMOST ALL DAYS
5. DON’T KNOW
6. PREFER NOT TO ANSWER

**MENT_Q4** In the last two weeks, how many days did you feel tired or with little energy?

1. NO DAYS
2. LESS THAN 1 WEEK
3. 1 WEEK OR MORE
4. ALMOST ALL DAYS
5. DON’T KNOW
6. PREFER NOT TO ANSWER

**MENT_Q5** In the last two weeks, how many days did you have lack of appetite or ate less?

1. NO DAYS
2. LESS THAN 1 WEEK
3. 1 WEEK OR MORE
4. ALMOST ALL DAYS
5. DON’T KNOW
6. PREFER NOT TO ANSWER

**MENT_Q6** In the last two weeks, how many days did you feel bad about yourself, thought you were a failure, or that you let down your family or yourself?

1. NO DAYS
2. LESS THAN 1 WEEK
3. 1 WEEK OR MORE
4. ALMOST ALL DAYS
5. DON’T KNOW
6. PREFER NOT TO ANSWER

**MENT_Q7** In the last two weeks, how many days did you have difficulty concentrating on things (such as reading a newspaper, watching, or listening to the radio)?

1. NO DAYS
2. LESS THAN 1 WEEK
3. 1 WEEK OR MORE
4. ALMOST ALL DAYS
5. DON’T KNOW
6. PREFER NOT TO ANSWER

**MENT_Q8** In the last two weeks, how many days did you feel slow in your movements or in speaking; or the contrary, in which you felt agitated and you stayed walking from one side to another, more than is customary?

1. NO DAYS
2. LESS THAN 1 WEEK
3. 1 WEEK OR MORE
4. ALMOST ALL DAYS
5. DON’T KNOW
6. PREFER NOT TO ANSWER

**MENT_Q9** In general, do you think you can make decisions by yourself, freely, without consulting your spouse or a family member? To which extent can you do this:

1. NO DAYS
2. SOMETIMES
3. ALMOST ALWAYS
4. ALWAYS
5. DON’T KNOW
6. PREFER NOT TO ANSWER

**Please rate the following statements as being either true, false, or you don’t know**.

|  | **TRUE (1)** | **FALSE (0)** | **DON’T KNOW (99)** |
| --- | --- | --- | --- |
| **ED_Q1** Teen pregnancy is an important issue in our community. |  |  |  |
| **ED_Q2** As a parent, I should be involved in my child’s development. |  |  |  |
| **ED_Q3** Contraception should be available for teens. |  |  |  |
| **ED_Q4** It is okay for a girl to be married after she completes primary education. |  |  |  |
| **ED_Q5** A girl should get married if she becomes pregnant while in school. |  |  |  |

**[If respondent has a child 5 - 19 years old in the house, CONTINUE, otherwise** → **SKIP to ECON_Q1]**

**ED_Q6** Now we are going to talk about schools and school children. Are any of your school-aged children currently *not* attending school, for more than the last three months?

1. YES
2. NO **→** **SKIP to ED_Q9**

**ED_Q7** What are the reasons for your child/children not being in school? **[Select all that apply]**

1. FEMALE CHILD IS PREGNANT OR HAS A BABY
2. LACK OF FINANCIAL RESOURCES
3. MALE CHILD IMPREGNATED SOMEONE
4. CHILD GOT MARRIED
5. CHILD GOT A JOB / EMPLOYMENT
6. CHILD REFUSED TO ATTEND
7. SCHOOL/TEACHER TURNED CHILD AWAY
8. OTHER, SPECIFY___________________________________________________

**[If respondent answered “FEMALE CHILD IS PREGNANT OR HAS A BABY”, CONTINUE, otherwise, →** **SKIP to ED_Q9]**

**ED_Q8** Does your daughter plan to re-enroll after delivery or when the baby is older?

1. YES
2. NO, DUE TO EMBARASSMENT OR STIGMA
3. NO, DUE TO DEMANDS OF CARING FOR A CHILD
4. NO, SHE WILL BE TOO OLD
5. NO, SHE IS NOT ALLOWED TO RE-ENROLL BY SOMEONE IN THE FAMILY
6. NO, SCHOOL/TEACHER TURNED CHILD AWAY
7. NO, OTHER, SPECIFY_______________________________
8. DON’T KNOW

**[If respondent has at least one boy 13 - 19 years old in the house, CONTINUE, otherwise →** **SKIP to ED_Q11]**

**ED_Q9** Have you ever talked to your *sons* of adolescent age about contraceptive methods, such as condoms, IUD, injections, pills, implants?

1. YES
2. NO
3. DON’T KNOW

**ED_Q10** From the topics below, tell me those you have discussed with your *sons* of adolescent age? **[Read the list][Select all that apply]**

1. PHYSICAL CHANGES
2. PUBERTY
3. PREGNANCY/ HAVING BABIES
4. PREVENTION OF STIs
5. PREVENTION OF HIV/AIDS
6. ABSTINENCE
7. ABORTION
8. USE OF CONTRACEPTIVES/FAMILY PLANNING
9. CONSEQUENCES OF PREMARITAL SEX
10. VIOLENCE AGAINST GIRLS OR WOMEN
11. SEXUAL ABUSE

[**If respondent has at least one girl 13 - 19 years old in the house, CONTINUE, otherwise →** **SKIP to ECON_Q1]**

**ED_Q11 H**ave you ever talked to your *daughters* of adolescent age about contraceptives, such as condoms, IUD, injections, pills, implants?

1. YES
2. NO
3. DON’T KNOW

**ED_Q12** From the topics below, tell me those you have discussed with your *daughters* of adolescent age? **[Read the list] [Select all that apply]**

1. PHYSICAL CHANGES
2. PUBERTY
3. MENSTRUATION
4. PREGNANCY/ HAVING BABIES
5. PREVENTION OF STIs
6. PREVENTION OF HIV/AIDS
7. ABSTINENCE
8. ABORTION
9. USE OF CONTRACEPTIVES/FAMILY PLANNING
10. CONSEQUENCES OF PREMARITAL SEX
11. VIOLENCE AGAINST GIRLS OR WOMEN
12. SEXUAL ABUSE

**ECON_Q1** Now I am going to ask you about your family’s business activities and finances. This is to help Lwala Community Alliance develop better programs for economic empowerment. All of this information will be kept completely confidential. Who is the main bread winner for your household?

1. NONE
2. I DO (RESPONDENT)
3. HUSBAND OR PARTNER
4. CO-WIFE
5. PARENT(S)
6. SIBLING(S)
7. RESPONDENT’S CHILDREN
8. RESPONDENT’S GRANDCHILDREN
9. CO-WIVES CHILDREN
10. AUNT OR UNCLE
11. GRANDPARENT
12. FRIEND
13. OTHER, SPECIFY_____________________________________________________

**ECON_Q2** Think about the *main source* of income for the household, what is the source of income? **[Read the list if necessary]**

1. EMPLOYED BY GOVERNMENT
2. EMPLOYED BY PRIVATE BUSINESS
3. EMPLOYED BY LWALA COMMUNITY ALLIANCE
4. PAID LABOUR IN PRIVATE AGRICULTURE
5. CASUAL LABOR
6. OWN AGRICULTURE
7. OWN LIVESTOCK BREEDING, ANIMAL PRODUCTS (CATTLE, GOATS, SHEEP)
8. OWN SMALL SCALE ANIMAL (POULTRY, FISH, RABBITS)
9. SELF-EMPLOYED
10. PENSIONER
11. INVESTMENTS
12. REMITTANCES
13. OTHER, SPECIFY__________________________________________________
14. DON’T KNOW

**ECON_Q3** Think about the *secondary source* of income for the household, what is the source of income?

1. EMPLOYED BY GOVERNMENT
2. EMPLOYED BY PRIVATE BUSINESS
3. EMPLOYED BY LWALA COMMUNITY ALLIANCE
4. PAID LABOUR IN PRIVATE AGRICULTURE
5. CASUAL LABOR
6. OWN AGRICULTURE
7. OWN LIVESTOCK BREEDING, ANIMAL PRODUCTS (CATTLE, GOATS, SHEEP)
8. OWN SMALL SCALE ANIMAL (POULTRY, FISH, RABBITS)
9. SELF-EMPLOYED
10. PENSIONER
11. INVESTMENTS
12. REMITTANCES
13. OTHER, SPECIFY__________________________________________________
14. DON’T KNOW

**ECON_Q4** On average, how much income does the household earn from *all sources* of income in one month *OR* one year? **[Remind them to consider all sources of income, such as selling, remittances, farming income and help them to calculate the total]**

1. MONTHLY INCOME, WRITE NUMBER (KES)____________
2. ANNUAL INCOME, WRITE NUMBER (KES)____________
3. DON’T KNOW
4. PREFER NOT TO ANSWER

**ECON_Q5** Think about this time last year compared to now, do you feel that your household is better off, worse off or the about the same, in terms of finances?

1. BETTER OFF
2. WORSE OFF
3. ABOUT THE SAME

99. DON’T KNOW

**ECON_Q6** Do you own any of the following items in your household? **[Read each item] [Select all that apply]**

1. RADIO
2. TELEVISION
3. REFRIGERATOR
4. IRON (CHARCOAL OR ELECTRIC)
5. KITCHEN SINK
6. ONE CELL PHONE **[if more than one, only select next option]**
7. TWO OR MORE CELL PHONES
8. COMPUTER OR LAPTOP
9. BICYCLE
10. MOTORBIKE
11. CAR
12. LAND, WITH TITLE DEED
13. WELL FOR WATER, IN THE YARD
14. POULTRY OR RABBITS
15. LIVESTOCK (SUCH AS COWS, GOATS, PIGS)

**ECON_Q7** How many towels does your household own?

1. NONE
2. ONE
3. TWO OR MORE

66. PREFER NOT TO ANSWER

99. DON’T KNOW

**ECON_Q8** How many frying pans does your household own?

1. NONE
2. ONE
3. TWO OR MORE

66. PREFER NOT TO ANSWER

99. DON’T KNOW

**ECON_Q9** Do you or a member of the house have a bank savings account? I do not want to know the exact institution or group you are a part of, just the types of savings accounts. **[Read the list] [Select all that apply]**

1. BANK (SUCH AS COOPERATIVE, KCB, OR EQUITY)
2. MOBILE BANKING (SUCH AS MPESA OR MSHWARI)
3. GROUP SAVINGS
4. NO BANK ACCOUNT, **→** **SKIP to ECON_Q11**
5. DON’T KNOW, **→** **SKIP to ECON_Q11**
6. PREFER NOT TO ANSWER, **→ SKIP to ECON_Q11**

**ECON_Q10** About how much money have you saved in all accounts?

1. LESS THAN 1,000 (KES)
2. 1,000 – 10,000
3. 10,001- 25,000
4. 25,001 - 50,000
5. 50,001 - 100,000
6. 100,001 - 300,000
7. 300,001 - 500,000
8. MORE THAN 500,000 (KES)
9. DON’T KNOW
10. PREFER NOT TO ANSWER

**ECON_Q11** Do you regularly participate in a table banking group? **[Explain table banking if unclear]**

1. YES
2. NO
3. DON’T KNOW
4. PREFER NOT TO ANSWER

**ECON_Q12** Do you have any loans from the following institutions? I do not want to know any information about what specific institutions you might have loans from, just the types of institutions. **[Read the list] [Select all that apply]**

1. NO CURRENT LOANS, **→** **SKIP to ECON_Q15**
2. TABLE BANKING
3. GROUP BANK
4. INDIVIDUAL PERSON
5. MICROFINANCE INSTITUTION
6. OTHER, SPECIFY____________________________________________________
7. DON’T KNOW, **→** **SKIP to ECON_Q15**
8. PREFER NOT TO ANSWER, **→** **SKIP to ECON_Q15**

**ECON_Q13** What is the highest interest rate on your current loans?

WRITE PERCENTAGE (%) ___________ **[Should be between 0% and 25%]**

1. DON’T KNOW
2. PREFER NOT TO ANSWER

**ECON_Q14** What is your main reason for borrowing money? **[Select all that apply]**

1. FEED AND CLOTHE FAMILY
2. SCHOOL FEES
3. ILLNESS/MEDICAL COSTS
4. SMALL BUSINESS
5. FARMING OR LIVESTOCK
6. BUILD/IMPROVE MY HOUSE
7. FUNERAL
8. WEDDING OR OTHER CEREMONY
9. OTHER, SPECIFY_________________________________________________
10. DON’T KNOW

**ECON_Q15** If you received additional income, what are two priorities you would spend money on? **[Read the list][Select only two priorities from the list]**

1. FOOD
2. CLOTHES
3. REPAIR/ EXPAND HOUSE
4. HEALTH SERVICES
5. SCHOOL FEES
6. WATER OR WATER-RELATED EQUIPMENT (TANK, GUTTERS)
7. ELECTRICITY SUPPLY
8. BUY CAR OR MOTORBIKE
9. OPEN OR EXPAND SHOP/BUSINESS
10. START PROFESSIONAL TRAINING
11. HIRE FARM STAFF
12. BUY LIVESTOCK GOODS/EQUIPMENT
13. BUY AGRICULTURAL GOODS/EQUIPMENT
14. RENT OR BUY FARM LAND
15. GREENHOUSE
16. ADD TO SAVINGS ACCOUNT
17. OTHER, SPECIFY________________________________________________
18. DON’T KNOW

**ECON_Q16** Do you currently have an active kitchen garden?

1. YES
2. NO, **→** **SKIP to ECON_Q22**

**ECON_Q17** I am going to list some common crops grown in a kitchen garden. Please tell us if you have grown the following crops in the last year. **[Select all that apply]**

1. TRADITIONAL VEGETABLES (SUCH AS APOTH, BOO, DEK, OSUGA)
2. EXOTIC VEGETABLES (KALES/SUKUMA)
3. NUTS/NJUNGU OR BEANS
4. HIGH VALUE CROPS (SUCH AS HO HO, KITUNGA, BEET ROOT, KARAT, DANIA, MORINGA, ROSELLE)
5. DON’T KNOW

**ECON_Q18** Do you grow crops from your kitchen garden for food only, food & income, or income only?

1. FOOD ONLY
2. FOOD AND INCOME
3. INCOME ONLY
4. DON’T KNOW

**ECON_Q19** How many meals do you serve from your kitchen garden in a week?

WRITE NUMBER OF MEALS____________

1. DON’T KNOW

**ECON_Q20** Do you practice irrigation in your kitchen garden at any time of the year?

1. YES
2. NO, **→** **SKIP to ECON_Q22**

**ECON_Q21** Which type of irrigation do you use in your kitchen garden at any time of the year? **[Read all options] [Select all that apply]**

1. DRIP
2. WATER CAN
3. FOOT PUMP
4. MECHANIZED/ENGINE PUMP
5. OTHER, SPECIFY_______________________________________________

**ECON_Q22** Do you or any member of your household own or rent agricultural land that you regularly farm?

1. YES, OWN
2. YES, OWN AND RENT
3. YES, RENT, **→** **SKIP to ECON_Q27**
4. NO

**ECON_Q23** What is the size of the agricultural land your household owns, in square acres?

WRITE ACRES______________ **[Okay to write parts of an acre, like 1.5]**

99. DON’T KNOW

**ECON_Q24** Which crops do you grow on your own farm land? **[Do not prompt] [Select all that apply]**

1. MAIZE
2. SUGARCANE
3. CASSAVA
4. SWEET POTATO
5. KALES
6. BEANS
7. FRUIT TREES (SUCH AS AVACADOS, PAPAYAS, OR BANANAS)
8. TREES (NON-FOOD)
9. OTHER, SPECIFY________________________________________________

**ECON_Q25** Do you practice irrigation in your farm at any time of the year?

1. YES
2. NO, **→** **SKIP to ECON_Q27**

**ECON_Q26** Which type of irrigation do you use in your farm? **[Read all options] [Select all that apply]**

1. DRIP
2. WATER CAN
3. FOOT PUMP
4. MECHANIZED/ENGINE PUMP
5. OTHER, SPECIFY________________________________________________

**ECON_Q27** Have you or any member of your household received agricultural training?

1. YES
2. NO, **→ SKIP to LCA_Q1**
3. DON’T KNOW, **→ SKIP to LCA_Q1**

**ECON_Q28** From which organization did you receive agriculture training? **[Select all that apply]**

1. MINISTRY OF AGRICULTURE
2. OTHER GOVERNMENT AGENCY
3. DIG (THROUGH LWALA)
4. FARMERS UNITED
5. OTHER NON-GOVERNMENT ORGANIZATION
6. OTHER, SPECIFY___________________________________________
7. DON’T KNOW

**LCA_Q1** Now I’m going to talk about something else. Have *you* ever visited the Lwala Community Hospital for any clinical service for yourself, (not including taking your child or family member)? For example, antenatal care, HIV testing or care, inpatient care, outpatient care, or other treatment.

1. YES
2. NEVER HEARD OF LWALA COMMUNITY HOSPITAL **→** **SKIP to LCA_Q5**
3. NO
4. DON’T KNOW

**LCA_Q2** **[If** **respondent is not married, → SKIP to LCA_Q3]** Has your *partner/spouse* ever visited the Lwala Community Hospital for any clinical service for him/herself (not including taking a child or family member to the Hospital)?

1. YES
2. NO
3. DON’T KNOW

**LCA_Q3 [If a child under 18 lives in the house, CONTINUE, otherwise, → SKIP to LCA_Q4]** Have any of your children received any clinical service (aside from delivery) from the Lwala Community Hospital? This includes immunizations or sickness. **[Any child in the household]**

1. YES
2. NO
3. DON’T KNOW

**LCA_Q4** Think about the last 3 months, how many times have you and members of your family visited the Lwala Community Hospital for any clinical service? **[add total for all members of the household]**

WRITE ESTIMATED TOTAL NUMBER OF VISITS__________

1. DON’T KNOW

**LCA_Q5** Now I am going to talk about something else. Lwala Community Alliance provides a variety of services and programs to people in North Kamagambo. Think about yourself and current members of your household. What Lwala Community Alliance services or programs have you or someone in your household participated in *within the last 1 year*? **[Select all that apply]**

1. NONE, **→ SKIP to LCA_Q7**
2. HOSPITAL SERVICES - INPATIENT
3. HOSPITAL SERVICES - ANC/DELIVERY
4. HOSPITAL SERVICES - CHILD WELFARE (SUCH AS IMMUNIZATIONS)
5. HOSPITAL SERVICES - OTHER OUTPATIENT
6. VISITED BY CHW EACH MONTH
7. FARMING GROUP PARTICIPANT
8. PUPIL IN EREADER PROGRAM
9. PUPIL IN HEALTH CLUB
10. PUPIL IN GIRLS MENTORING
11. GIRL RECEIVED PADS
12. GIRL RECEIVED UNIFORMS
13. YOUTH ATTENDED BETTER BREAKS
14. YOUTH ATTENDING YOUTH FRIENDLY CORNER
15. YOUTH IN OUT-OF-SCHOOL HEALTH CLUB
16. YOUTH IS YPP
17. SUPPORT GROUP PARTICIPANT
18. TABLE BANKING GROUP PARTICIPANT WITH SUPPORT FROM LCA/DIG
19. OUT OF SCHOOL GIRLS MENTORING PARTICIPANT
20. SECONDARY SCHOOL SPONSORSHIP
21. KIVA LOAN
22. WASH TRAINED
23. VISITED BY WASH TEAM (CLTS)
24. ATTENDED COMMUNITY OUTREACH
25. ATTENDED COMMUNITY DIALOGUE
26. ATTENDED WASH TOURNAMENT
27. TRAINED ON CHILD NUTRITION
28. PARENT’S CLUB PARTICIPANT
29. ATTENDED OTHER TRAINING AT LCA
30. NEW VISIONS MEMBER
31. KANGU PARTICIPANT
32. OTHER, SPECIFY______________________________________________
33. YES, BUT DON’T KNOW WHAT, **→ SKIP to LCA_Q7**

**LCA_Q6** Of the programs and services you or your family has participated in, which has the most value for you? **[Select only one from previous list]**

WRITE LETTER FROM PREVIOUS LIST_____________

**LCA_Q7** Have you ever recommended a Lwala Community Alliance service or program to a friend or neighbor? **[If unsure, ask if they’ve ever told a friend or neighbor to go to Lwala for assistance]**

1. YES
2. NO
3. DON’T KNOW

**LCA_Q8** If you could, what program or service would you most like to get involved in at Lwala Community Alliance next?

CHOOSE FROM OPTIONS ABOVE AND WRITE LETTER____________

88. OTHER, SPECIFY

1. DON’T KNOW

**LCA_Q9** Mention three services or programs that LCA does not currently offer that you wish they would.

1. _________________________________________
2. _________________________________________
3. _________________________________________

**[If there is a child under 5 years of age in the home, CONTINUE, otherwise,** → **SKIP to OBV_Q1**

**VACC_Q1** Now I have a few questions about the youngest of your children (last born). Do you have the Maternal Child Health Book where the child’s vaccinations are written down?

1. YES, **[ask to see it and complete vaccination table]**
2. NOT AVAILABLE (LOST, DESTROYED, NOT HERE) → **SKIP to OBV_Q1**
3. NEVER HAD A BOOK → **SKIP to OBV_Q1**
4. DON’T KNOW → **SKIP to OBV_Q1**
5. PREFER NOT TO SHOW IT → **SKIP to OBV_Q1**

**Record whether the child has been immunized for each of the following immunizations:**

**VACC_Q2** BCG

1. YES
2. NO

**VACC_Q3** POLIO 0

1. YES
2. NO

**VACC_Q4** POLIO 1

1. YES
2. NO

**VACC_Q5** POLIO 2

1. YES
2. NO

**VACC_Q6** POLIO 3

1. YES
2. NO

**VACC_Q7** DPT 1

1. YES
2. NO

**VACC_Q8** DPT 2

1. YES
2. NO

**VACC_Q9** DPT 3

1. YES
2. NO

**VACC_Q10** MEASLES

1. YES
2. NO

**VACC_Q11** VITAMIN A

1. YES
2. NO

**VACC_Q12** PNEUMOCOCCAL 1

1. YES
2. NO

**VACC_Q13** PNEUMOCOCCAL 2

1. YES
2. NO

**VACC_Q14** PNEUMOCOCCAL 3

1. YES
2. NO

**[Observation Section] Read -** Thank you. We are done with these questions. Now I’d like to ask you to show me a few things and then we will be done.

**OBV_Q1** Did you sleep under a mosquito net last night?

1. YES
2. NO **→ SKIP to OBV_Q3**
3. DON’T KNOW **→ SKIP to OBV_Q3**

**OBV_Q2** Can you show me the mosquito net you slept under?

1. YES, GOOD QUALITY NET
2. YES, POOR QUALITY NET OBSERVED
3. YES, NO NET OBSERVED
4. OTHER, SPECIFY________________________
5. NO PERMISSION TO SEE

**OBV_Q3** Did **[child’s name]** sleep under a mosquito net last night?

1. YES
2. NO **→ SKIP to OBV_Q5**
3. DON’T KNOW **→ SKIP to OBV_Q5**

**OBV_Q4** Can you show me the mosquito net that **[child’s name]** slept under?

1. YES, SAME NET AS THE RESPONDENT (GOOD QUALITY)
2. YES, SAME NET AS THE RESPONDENT (POOR QUALITY)
3. YES, DIFFERENT NET AS THE RESPONDENT (GOOD QUALITY)
4. YES, DIFFERENT NET AS THE RESPONDENT (POOR QUALITY)
5. YES, NO NET OBSERVED
6. OTHER, SPECIFY________________________
7. NO PERMISSION TO SEE

**OBV_Q5** Who in this household normally sleeps under mosquito nets? **[Select all that apply.]**

1. THE MAN OF THE HOUSE
2. YOU
3. THE CHILDREN
4. EVERYONE
5. OTHERS, SPECIFY________________________________
6. DON’T KNOW

**OBV_Q6** How many mosquito nets does your family have in this house? **[Read the options]**

1. NONE **→** **SKIP to OBV_Q12**
2. LESS THAN THE NUMBER OF BEDS/MATS
3. ONE FOR EVERY BED/MAT
4. MORE THAN THE NUMBER OF BEDS/MATS]
5. DON’T KNOW **→** **SKIP to OBV_Q12**

**OBV_Q7** How many months ago did you obtain the newest mosquito net? **[If less than 1 month, 2 weeks for example, mark 1 month]**

YEARS____________MONTHS____________

1. DON’T KNOW

**OBV_Q8** How many of the nets in your household were donated?

NUMBER______________

1. DON’T KNOW

**OBV_Q9** How many of the nets in your household were purchased?

NUMBER______________

1. DON’T KNOW

**OBV_Q10** Do you need to treat any of the nets in the household with insecticide?

1. YES
2. NO **→ SKIP to OBV_Q12**
3. DON’T KNOW **→ SKIP to OBV_Q12**

**OBV_Q11** Which one needs to be soaked?

1. A DONATED NET
2. A PURCHASED NET
3. BOTH
4. DON’T KNOW

**OBV_Q12** Is there a season of the year when you do NOT need to use mosquito nets? **[Select all that apply]**

1. NO
2. YES, DRY SEASON
3. YES, SHORT RAINS
4. YES, LONG RAINS
5. DON’T KNOW

**OVV_Q13** How many habitable rooms does this household occupy in its main dwelling (do not count storerooms, toilets, bathrooms)?

1. ONE
2. TWO
3. THREE
4. FOUR
5. FIVE
6. SIX OR MORE

**OBV_Q14** The floor of the main dwelling is predominantly made of what material?

1. EARTH OR WOOD
2. CEMENT OR TILES
3. OTHER

66. PREFER NOT TO ANSWER

**OBV_Q15** What is the main source of lighting fuel for the household?

1. COLLECTED FIREWOOD, PURCHASED FIREWOOD, GRASS OR DRY CELL (TORCH)
2. PARAFFIN, CANDLES OR BIOGAS
3. ELECTRICITY
4. SOLAR OR GAS
5. OTHER

66. PREFER NOT TO ANSWER

99. DON’T KNOW

**[If the respondent has a kitchen garden, CONTINUE, otherwise → SKIP to OBV_Q15]**

**OBV_Q16** Can you show me your kitchen garden? **[Measure the kitchen garden in steps]**

WRITE SIZE __________ STEPS by __________ STEPS **[1 step = 1 meter]**

1. NO PERMISSION TO SEE

**OBV_Q17 [Mark whether the kitchen garden has enriched, raised beds.]**

1. YES
2. NO
3. NO PERMISSION TO SEE

**OBV_Q18** Can you please show me where members of your household most often wash their hands?

1. OBSERVED, FIXED PLACE
2. OBSERVED, MOBILE
3. NOT OBSERVED, NOT IN DWELLING/YARD, **→** **SKIP to OBV_Q21**
4. OTHER, SPECIFY____________________________________
5. NO PERMISSION TO SEE, **→** **SKIP to OBV_Q21**

**Observe presence of water and soap at the place for hand washing and record observation.**

**OBV_Q19** Water

1. WATER IS AVAILABLE
2. WATER IS NOT AVAILABLE

**OBV_Q20** Soap

1. SOAP IS AVAILABLE
2. SOAP IS NOT AVAILABLE

**OBV_Q21** Can you now show me where you put your dishes after washing them?

1. OUTSIDE DRYING RACK
2. OUTSIDE ON THE GROUND
3. INSIDE ON A RACK OR TABLE
4. INSIDE ON THE GROUND
5. IN A BUCKET OR BASIN
6. NOT OBSERVED
7. OTHER, SPECIFY_______________________________________________
8. DON’T KNOW
9. NO PERMISSION TO SEE

**OBV_Q22** I’d like to take a look at where you cook – can you show me that place?

1. OUTDOORS
2. SEPARATE KITCHEN, VENTILATED
3. SEPARATE KITCHEN, NOT VENTILATED
4. IN THE HOME, VENTILATED
5. IN THE HOME, NOT VENTILATED
6. NO PERMISSION TO SEE

**OBV_Q23** What fuel do you usually use when you cook?

1. FIREWOOD, OPEN STOVE
2. FIREWOOD, IMPROVED STOVE
3. CHARCOAL/JIKO
4. PARAFIN
5. GAS STOVE
6. ELECTRIC STOVE
7. OTHER, SPECIFY_______________________________________________
8. DON’T KNOW

**OBV_Q24** Currently, what kind of toilet facility does you and members of your household use?

1. NO FACILITY/BUSH/FIELD/SUGARCANE PLANTATION, **→** **SKIP to End of Survey**
2. TRADITIONAL PIT TOILET
3. VENTILATED IMPROVED PIT LATRINE
4. NEIGHBOR’S TRADITIONAL PIT TOILET
5. NEIGHBOR’S IMPROVED PIT LATRINE
6. OTHER, SPECIFY ________________________________,

**OBV_Q25** Can you show me the latrine? **[Observe the condition of the latrine and record observation.]**

1. NOT OBSERVED
2. GOOD CONDITION
3. POOR BUT USABLE
4. NOT USABLE / CLOSED UP
5. OTHER, SPECIFY________________________________________________
6. NO PERMISSION TO SEE

**[END OF SURVEY]**

**[Note time of completion]:**  ______11:14 AM__________ AM / PM

**Read** - That is the end of the survey. Thank you for your participation.

Please remember that your responses will be kept confidential and will not be shared with any of your family, friends, or neighbors. If you have any questions about the Lwala Community Alliance, please contact us through the information provided in the consent form you were given.

The responses you have provided will help Lwala Community Alliance improve the services that are available to you.

Do you have any questions for me about the survey?

**[Give respondent airtime, and record amount given]:** ____200/=_______shillings
