# Supplementary material for: Under-five mortality in the Rongo Sub-County of Migori County, Kenya: Experience of the Lwala Community Alliance 2007-2017 with evidence from a cross-sectional survey
Source: PLoS One. 2018 Sep 7;13(9):e0203690. doi: 10.1371/journal.pone.0203690 (PMC6128651; doi:10.1371/journal.pone.0203690)
Supplement: S2 Appendix — An additional model that included child gender did not differ substantially in hazard ratios or significance compared to the model presented in the main body of the manuscript. (DOCX) [file pone.0203690.s002.docx]

| **S2 Table. Multivariable Cox Model for Association with Under-Five Mortality Including Child Gender.** | | | |
| --- | --- | --- | --- |
| N = 1,359 | Hazard Ratio | 95% CI | P-value |
| Year of Birth | **0.931** | **(0.876,0.988)** | **0.019** |
| Child Gender | 1.094 | (0.740,1.617) | 0.654 |
| Maternal Age | 0.979 | (0.918,1.044) | 0.52 |
| Respondent Currently Married/In Relationship | 2.603 | (0.737,9.194) | 0.137 |
| Household has Cell Phone | 0.859 | (0.521,1.418) | 0.553 |
| Improved Pit Latrine in House | 0.48 | (0.177,1.303) | 0.15 |
| Household has Livestock | 1.597 | (0.877,2.906) | 0.126 |
| Born During Long Rain Season | **1.973** | **(1.342,2.900)** | **0.001** |
| Time Since Previous Birth > 18 months | **0.343** | **(0.201,0.584)** | **<0.001** |
| Multiple-Gestation Pregnancy | **6.218** | **(2.081,18.578)** | **0.001** |

**S2 Appendix. Additional model with child gender.**

An additional model that included child gender did not differ substantially in hazard ratios of significance compared to the model presented in the main body of the manuscript.
